# Cardiovascular risk factors and the allostatic interoceptive network in dementia

**DOI:** 10.1093/cvr/cvaf185

**Published:** 2025-10-10

**Authors:** Jessica L Hazelton, Joaquín Migeot, Raul Gonzalez-Gomez, Florencia Altschuler, Claudia Duran-Aniotz, Olivia Wen, Dante Sebastián Galván Galván Rial, Pablo Barttfeld, Vicente Medel, Cecilia González Campo, Ana María Castro-Laguardia, Hernán Hernández, Carolina Gonzalez-Silva, Olga Castaner, Kun Hu, Peng Li, María Isabel Behrens, Martin A Bruno, Juan Felipe Cardona, Nilton Custodio, Hernando Santamaria-Garcia, Adolfo M Garcia, Maria E Godoy, José Alberto Avila-Funes, Marce Maito, Diana L Matallana, Bruce Miller, Francisco Lopera, Maira Okada de Oliveira, Stefanie D Pina-Escudero, Katherine L Possin, Elisa de Paula France Resende, Pablo Reyes, Andrea Slachevsky, Ana Luisa Sosa, Leonel T Takada, Jennifer S Yokoyama, Agustin Ibanez

**Affiliations:** Latin American Brain Health Institute (BrainLat), Universidad Adolfo Ibáñez, Santiago, Chile; Cognitive Neuroscience Center (CNC), Universidad de San Andrés, Buenos Aires, Argentina; The University of Sydney, Brain and Mind Centre, School of Psychology, Sydney, Australia; Latin American Brain Health Institute (BrainLat), Universidad Adolfo Ibáñez, Santiago, Chile; Global Brain Health Institute (GBHI), Trinity College Dublin, Dublin, Ireland; Latin American Brain Health Institute (BrainLat), Universidad Adolfo Ibáñez, Santiago, Chile; Latin American Brain Health Institute (BrainLat), Universidad Adolfo Ibáñez, Santiago, Chile; Cognitive Neuroscience Center (CNC), Universidad de San Andrés, Buenos Aires, Argentina; Latin American Brain Health Institute (BrainLat), Universidad Adolfo Ibáñez, Santiago, Chile; Global Brain Health Institute (GBHI), Trinity College Dublin, Dublin, Ireland; Latin American Brain Health Institute (BrainLat), Universidad Adolfo Ibáñez, Santiago, Chile; Cognitive Science Group, Instituto de Investigaciones Psicológicas (IIPsi, CONICET-UNC), Facultad de Psicología, Universidad Nacional de Córdoba, Córdoba, Argentina; Latin American Brain Health Institute (BrainLat), Universidad Adolfo Ibáñez, Santiago, Chile; Cognitive Science Group, Instituto de Investigaciones Psicológicas (IIPsi, CONICET-UNC), Facultad de Psicología, Universidad Nacional de Córdoba, Córdoba, Argentina; Latin American Brain Health Institute (BrainLat), Universidad Adolfo Ibáñez, Santiago, Chile; Latin American Brain Health Institute (BrainLat), Universidad Adolfo Ibáñez, Santiago, Chile; Cognitive Neuroscience Center (CNC), Universidad de San Andrés, Buenos Aires, Argentina; Latin American Brain Health Institute (BrainLat), Universidad Adolfo Ibáñez, Santiago, Chile; Latin American Brain Health Institute (BrainLat), Universidad Adolfo Ibáñez, Santiago, Chile; Latin American Brain Health Institute (BrainLat), Universidad Adolfo Ibáñez, Santiago, Chile; Global Brain Health Institute (GBHI), Trinity College Dublin, Dublin, Ireland; Cardiovascular Risk and Nutrition Research Group, Hospital del Mar Research Institute, Spain; CIBER Epidemiology and Public Health (CIBERESP), ISCIII, Spain; Department of Anesthesia, Critical Care and Pain Medicine, Massachusetts General Hospital, Harvard Medical School, Boston, Massachusetts, USA; Department of Anesthesia, Critical Care and Pain Medicine, Massachusetts General Hospital, Harvard Medical School, Boston, Massachusetts, USA; Division of Sleep Medicine, Harvard Medical School, Boston, MA, USA; Division of Sleep and Circadian Disorders, Brigham and Women’s Hospital, Harvard Medical School, Boston, MA, USA; Departamento de Neurociencia, Facultad de Medicina, Universidad de Chile, Santiago, Chile; Departamento de Neurología y Neurocirugía, Hospital Clínico Universidad de Chile; Centro de Investigación Clínica Avanzada (CICA), Universidad de Chile, Santiago, Santiago de Chile, Chile; Servicio de Neurología, Departamento de Medicina, Clínica Alemana-Universidad del Desarrollo, Santiago de Chile, Chile; Instituto de Ciencias Biomédicas, Universidad Católica de Cuyo, San Juan, Argentina; Facultad de Psicología, Universidad del Valle, Cali, Colombia; Unit Cognitive Impairment and Dementia Prevention, Peruvian Institute of Neurosciences, Lima, Peru; Pontificia Universidad Javeriana, PhD Program of Neuroscience, Bogotá, Colombia; Hospital Universitario San Ignacio, Centro de Memoria y Cognición Intellectus, Bogotá, Colombia; Cognitive Neuroscience Center (CNC), Universidad de San Andrés, Buenos Aires, Argentina; Global Brain Health Institute (GBHI), Trinity College Dublin, Dublin, Ireland; Departamento de Lingüística y Literatura, Universidad de Santiago de Chile, Santiago, Chile; Latin American Brain Health Institute (BrainLat), Universidad Adolfo Ibáñez, Santiago, Chile; Cognitive Neuroscience Center (CNC), Universidad de San Andrés, Buenos Aires, Argentina; Dirección de Enseñanza, Instituto Nacional de Ciencias Médicas y Nutrición Salvador Zubirán, Mexico City, Mexico; Latin American Brain Health Institute (BrainLat), Universidad Adolfo Ibáñez, Santiago, Chile; Instituto de Envejecimiento, Facultad de Medicina, Pontificia Universidad Javeriana, Bogotá D.C., Colombia; Center for Memory and Cognition, Hospital Universitario San Ignacio Bogotá, San Ignacio, Bogotá D.C., Colombia; Global Brain Health Institute, University of California, San Francisco, California, USA; Department of Neurology, Memory and Aging Center, University of California, San Francisco, California, USA; Grupo de Neurociencias de Antioquia, University of Antioquia, Medellín, Colombia; Departamento de Lingüística y Literatura, Universidad de Santiago de Chile, Santiago, Chile; Global Brain Health Institute, University of California, San Francisco, California, USA; Global Brain Health Institute, University of California, San Francisco, California, USA; Department of Neurology, Memory and Aging Center, University of California, San Francisco, California, USA; Global Brain Health Institute, University of California, San Francisco, California, USA; Department of Neurology, Memory and Aging Center, University of California, San Francisco, California, USA; Global Brain Health Institute, University of California, San Francisco, California, USA; Universidade Federal de Minas Gerais, Belo Horizonte, Minas Gerais, Brazil; Instituto de Envejecimiento, Facultad de Medicina, Pontificia Universidad Javeriana, Bogotá D.C., Colombia; Faculty of Medicine, University of Chile, Santiago, Chile; Departamento de Neurología y Neurocirugía, Hospital Clínico Universidad de Chile; Neurology Department, Memory and Neuropsychiatric Center (CMYN), Hospital d el Salvador; Departamento de Medicina, Servicio de Neurología, Clínica Alemana-Universidad del Desarrollo, Santiago, Santiago de Chile, Chile; Laboratorio de Demencias del Instituto Nacional de Neurología y Neurocirugía Manuel Velasco Suárez, Mexico City, CDMX, México; Grupo de Neurociencias de Antioquia, University of Antioquia, Medellín, Colombia; Global Brain Health Institute, University of California, San Francisco, California, USA; Department of Neurology, Memory and Aging Center, University of California, San Francisco, California, USA; Department of Radiology and Biomedical Imaging, University of California, San Francisco, San Francisco, CA 94158, USA; Latin American Brain Health Institute (BrainLat), Universidad Adolfo Ibáñez, Santiago, Chile; Cognitive Neuroscience Center (CNC), Universidad de San Andrés, Buenos Aires, Argentina; Global Brain Health Institute (GBHI), Trinity College Dublin, Dublin, Ireland; Department of Biophysics, School of Medicine, Istanbul Medipol University, 34815 Istanbul, Türkiye

**Keywords:** Cardiovascular health, Aging, Dementia, Neurodegeneration, Cardiovascular risk, Brain atrophy, Functional connectivity

## Abstract

**Aims:**

Cardiovascular risk factors, such diabetes, hypertension, blood pressure, obesity, and smoking, are linked with allostatic-interoception—the continuous monitoring of internal bodily states in anticipation of environmental demands. These risk factors are associated with dementia risk. How these factors affect brain networks vulnerable to neurodegeneration and involved in allostatic-interoception, such as the Allostatic-Interoceptive Network (AIN), is unknown. We investigated the relationship between cardiovascular risk and AIN structure and function in frontotemporal lobar degeneration (FTLD) and Alzheimer’s disease (AD).

**Methods and results:**

We recruited 1501 participants (304 with FTLD, 512 with AD, and 685 healthy controls) from the Multi-Partner Consortium to Expand Dementia Research in Latin America (ReDLat). A cardiovascular risk score was calculated based on: age, sex, diabetes, hypertension, systolic blood pressure, body mass index, and smoking status. Cardiovascular risk was associated with grey matter integrity and functional connectivity in age- and sex-matched patient-control groups focusing on predefined regions of interest within the AIN. Higher cardiovascular risk was associated with reduced structural integrity and functional connectivity within the AIN in both FTLD and AD. FTLD patients showed more extensive structural and functional connectivity disruptions throughout the AIN. In AD patients, structural reductions in the AIN were prominent, with functional connectivity restricted to the hippocampus, parahippocampal gyrus, and orbitofrontal regions.

**Conclusion:**

Cardiovascular risk factors appear to adversely impact the AIN structure and function, with disease-specific patterns of vulnerability. Results underscore the importance of integrating cardiovascular health into models of neurodegenerative disease and managing cardiovascular health to support brain integrity in dementia.

**Time of primary review: 79 days**

**See the editorial comment for this article ‘Allostatic-interoceptive brain network: a bridge between cardiovascular burden and dementias’, by L. Carnevale, https://doi.org/10.1093/cvr/cvaf198.**

## 1. Introduction

Cardiovascular risk factors, including diabetes, hypertension, blood pressure, obesity, and smoking, are strongly associated with an increased risk of developing dementia^1^ contributing to neurodegeneration through heightened vascular burden.^2^ Emerging synergistic approaches to brain health and disease call for the integration of comorbidities such as cardiovascular risk factors in understanding dementia.^3,4^ Indeed, population-based studies indicate that individuals with cardiovascular comorbidities are five times more likely to develop all-cause dementia, with this risk being independent of Alzheimer’s disease (AD)-related genetic predispositions.^5^ However, the specific impact of cardiovascular risk factors on brain networks vulnerable to dementia remains poorly understood.

The predictive coding theory of allostatic interoception^6,7^ states that the brain anticipates and processes internal bodily signals to adapt to environmental demands.^6–13^ Allostatic overload arises when the body’s adaptive capacity is depleted over time due to chronic stress or environmental pressures.^6,7,11^ Cardiovascular function and allostatic-interoception are deeply interdependent.^14–16^ Higher allostatic load as measured by a composite score of cardiometabolic factors—such as blood pressure, body composition, cholesterol levels, and cortisol—has been linked to adverse aging outcomes.^17^ Despite the connection between cardiovascular risk and allostatic-interoception, no study has examined how cardiovascular risk factors influence brain networks particularly vulnerable to dementia.

Emerging evidence has shown that dysfunction in allostatic-interoception, encompassing behavioral, peripheral, and neural measures, are observed in frontotemporal lobar degeneration (FTLD) syndromes,^13,18–27^ particularly in behavioral variant frontotemporal dementia (bvFTD). While evidence for interoceptive impairment in AD is mixed (18, 19, but see 22), altered allostatic markers have been consistently reported.^26,28–31^ Allostatic-interoception is supported by the allostatic-interoceptive network (AIN), a large-scale brain network that includes cortical and subcortical structures (e.g. insula, anterior cingulate cortex, orbitofrontal cortex, amygdala, hippocampus, parahippocampus, and thalamus).^6,7,18^ Disruptions in the structural and functional integrity of the AIN have been documented in both FTLD and AD.^18^ However, the relationship between cardiovascular risk factors and changes in the AIN in dementia remains largely unexplored.

Taken together, this evidence suggests a hypothesis: cardiovascular risk factors may influence the AIN in dementia.^6–8,32,33^ Majority of studies to date, however, have predominantly focused on total grey matter volume,^32–34^ which does not provide a measure of specific brain structures associated with cardiovascular risk. Whilst some studies have focused on a limited subset of brain regions (e.g. hippocampus), these have been conducted in healthy adults or aging populations^32,33,35,36^ limiting their application to dementia populations. Critically, no study has investigated how cardiovascular risk factors may influence both structural and functional changes in the brain within AIN, a key network that we propose is related to cardiovascular risk. Further, no study has examined how cardiovascular risk factors contribute to neurodegeneration in dementia syndromes such as FTLD and AD, where allostatic-interoception dysfunction has been reported.^18–22,27^ Further, to our knowledge, this association has not been investigated in Latin America, where cardiovascular risk factors are increased in the general population.^37–39^ Understanding how cardiovascular risk factors may relate to underlying neural mechanisms in dementia syndromes will further refine our understanding of these diseases and may bolster future precision medicine approaches by reducing comorbid risk factors.

In the current study, we recruited a Latin American and US cohort (*N* = 1501) from the Multi-Partner Consortium to Expand Dementia Research in Latin America (ReDLat).^31,32^ We investigated cardiovascular risk factors (e.g. diabetes, hypertension, systolic blood pressure, body mass index (BMI), and current smoking status) using the non-laboratory-based Framingham’s Risk Score (FRS).^40^ We investigated the FRS alongside both structural and functional brain connectivity measures. Despite differences in allostatic-interoception and cardiovascular risk profiles in each dementia syndrome, we hypothesized that reduced structural and functional connectivity within the AIN would be associated with greater cardiovascular risk factors in both dementia syndromes, as evidence of an underlying neurobiological mechanism contributing to both processes.

## 2. Methods

### 2.1 Participants

**Figure 1:**
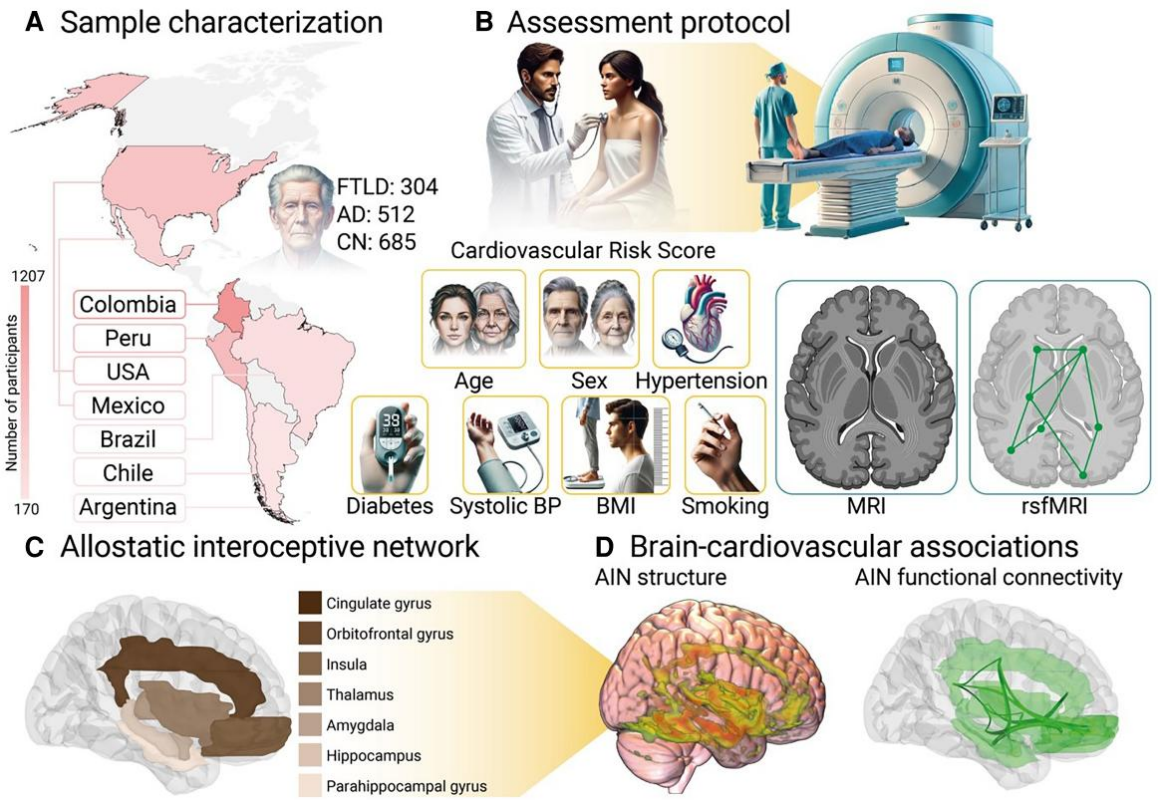
Experimental workflow. *A*) Sample characterization per country. Colour bars indicate the number of participants. Circle plots represent the proportion of participants in each group. *B*) Assessment protocol. All participants underwent a clinical examination and had an MRI scan. The Framingham Risk Score (FRS) was calculated based on established non-laboratory measures, considering Age, Sex, Presence of Diabetes, Presence of Hypertension, Systolic blood pressure, BMI, and current smoking status. MRI measures included structural T1 MRI and resting state functional MRI (rsFMRI). *C*). AIN regions investigated in the neuroimaging analyses. *D*) Brain-cardiovascular associations using the FRS were investigated in the AIN using structural VBM and functional connectivity analyses.

The experimental workflow is shown in *Figure 1*. We recruited 1501 participants, including 304 FTLD patients, 512 AD patients, and 685 healthy controls (CN) from both Latin America and the United States (see Supplementary material online, *Tables S1* and *S2* for subtype-specific information). All participants were recruited through ReDLat’s ongoing multicenter protocols^41,42^ involving clinical examination, neuropsychological testing, and magnetic resonance imaging (MRI). Patients were diagnosed with FTLD syndromes based on the current sets of diagnostic criteria, including prominent changes to behavior, personality and/or language,^43,44^ and motor features.^45–47^ AD syndrome was diagnosed based on current sets of diagnostic criteria, including typical AD with an amnestic profile^48^ and atypical variants based on language features,^43^ visual features,^49^ and behavior.^50^ All CNs scored >24 on the Mini Mental State Examination (MMSE).^51,52^ Exclusion criteria included presence of major primary cardiovascular compromise (i.e. cardiovascular disease), history of other neurological disorders, psychiatric conditions, or substance abuse. CNs were demographically matched (i.e. age and sex matched) to each patient group (FTLD or AD) using R MatchIt to create disease-control groups for comparison,^53^ due to demographic differences between patient groups and controls in the full dataset. Approximately 10% of AD cases were also removed during the matching process due to older age (>85 years of age). All participants or their caregivers provided informed consent in line with the Declaration of Helsinki. The study was approved by the Ethics Committees of the involved institutions.

### 2.2 Measures

#### 2.2.1 Cognition

The MMSE was used to measure cognitive performance based on measures of attention, memory, language, and visuospatial abilities.^52^ Total MMSE scores are out of 30, with higher scores representing better performance.

#### 2.2.2 Disease severity

Two measures of disease severity were calculated: (ⅰ) The Clinical Dementia Rating (CDR) in AD, and (ⅱ) the CDR scale—FTLD (CDR-FTLD) in FTLD. In brief, both measures assess functionality using a semi-structured interview with patients and their informants and cover six domains including memory, orientation, problem solving/judgment, community affairs, home and hobbies, and personal care.^54^ In addition, the CDR-FTLD includes measures of behavior relevant for FTLD patients.^55^ The CDR-SOB (Sum of Boxes) is calculated by summing each of the domain scores. Higher CDR-SOB scores represent greater functional impairment.

#### 2.2.3 Cardiovascular risk

The Framingham's Risk Score (FRS) was calculated based on non-laboratory to measure cardiovascular risk.^40^ This score is previously validated,^40^ and measures cardiovascular risk based on age, biological sex (assigned male or female at birth), BMI, systolic blood pressure, hypertension status (anti-hypertensive medication use and/or clinical report), diabetes status (diabetic medication and/or clinical report), and current smoking status^40^(see Supplementary material online, *Table S3*). High agreement between the non-laboratory-based and laboratory-based versions of the FRS has been reported.^56–59^ In brief, each measure is scored following previously validated guidelines, taking into account biological sex differences in cardiovascular risk.^40^ Higher FRS scores represent greater cardiovascular risk. Missing data were present in <10% of each variable necessary to calculate the FRS and were evenly distributed across groups. To handle missing data, we employed multiple imputation using the MICE (Multivariate Imputation by Chained Equations) package in R.^60^

### 2.3 Neuroimaging acquisition

Whole-brain structural MRI and resting-state functional MRI data were obtained, and standard pre-processing steps were followed as recommended by the Organization for Human Mapping.^61,62^ Each center followed standard protocols (see Supplementary material online, *Tables S4* and *S5* scanner details and acquisitions).

### 2.4 Statistical analyses

#### 2.4.1 Demographics

Demographic, neuropsychological, and cardiovascular risk variables were compared via *t*-tests (i.e. age, education, cognition, cardiovascular risk), or chi-square tests (i.e. biological sex). All behavioral analyses were conducted using Python (v.3.10.12) with Pandas package (v.2.0.3)^63^ and Statsmodel package (v.0.14.2).^64^

#### 2.4.2 Voxel-based morphometry

Voxel-based morphometry (VBM) was performed using the Computational Anatomy Toolbox (CAT12, https://neuro-jena.github.io/cat/) in MATLAB R2022a. Standard pre-processing steps were followed, including bias-field correction, noise reduction, skull stripping, segmentation, and normalization to the Montreal Neurological Institute (MNI) space with a resolution of 1.5 isotropic, using default parameters. Sample homogeneity and orthogonality checks were performed. Regions of interest (ROI) masks were created using the MarsBar toolbox^65^ for the AIN (insula, anterior cingulate cortex, mid-cingulate cortex, orbitofrontal cortex, amygdala, hippocampus, parahippocampus, and thalamus)^7,8,18^ using the Automated Anatomical Labeling (AAL-2) atlas.^66^ Pearson correlations were conducted between TIV-corrected GM volume and cardiovascular risk. Within the AIN, regression analyses were conducted with the FRS score, controlling for group (FTLD vs. CN; AD vs. CN), scanner, and total intracranial volume. To directly compare our AD and FTLD groups, we transformed our pre-processed data by transforming the normalized and smoothed outputs to w-scored images.^67–70^ Here, w-scores (Mean = 0, Standard deviation = 1) show how different the observed GM volume in each voxel is (e.g. positive or negative w-score) than expected, based on an individual’s global composite score adjusted for specific covariates (e.g. age, sex, diagnosis, total intracranial volume, and scanner type). This approach has been previously used in neurodegenerative studies to account for demographic differences and scanner effects without losing information regarding diagnostic effects.^67–70^ The resulting w-score maps of each individual were used for the direct comparison between AD and FTLD. Here, regression analyses were conducted with the FRS score and the interaction between FRS score and diagnosis (AD vs. FTLD) was entered into the model. All clusters are reported using threshold-free cluster enhancement, at FDR-corrected, *P* < 0.05 with a contiguous threshold of 50 voxels.

#### 2.4.3 Resting-state functional connectivity

All data were pre-processed following a standard pipeline in CONN (22.a)^60,61^ using SPM (v.12)^71^(see Supplementary methods). In brief, pre-processing steps involved spatial convolution smoothing with a Gaussian kernel of 6 mm full width half maximum (FWHM). Next, functional data were denoised using a standardized denoising pipeline in CONN.^72^ We focused our analyses on ROI-to-ROI functional connectivity between regions within the AIN,^7,8,18^ mirroring the masks outlined in our structural analyses for comparison (see Supplementary material for further details). Group-level analyses were performed using a General Linear Model (GLM).^72^ For each individual connection a separate GLM was estimated, with first-level connectivity measures at this connection as dependent variables, and FRS as independent variable, with scanner and group as a covariate. Connection-level hypotheses were evaluated using multivariate parametric statistics with random-effects across subjects and sample covariance estimation across multiple measurements. Inferences were performed at the level of individual clusters (groups of similar connections). Cluster-level inferences were based on parametric statistics within- and between- each pair of networks (Functional Network Connectivity),^73^ with networks identified using a complete-linkage hierarchical clustering procedure based on ROI-to-ROI anatomical proximity and functional similarity metrics.^72^ Results were reported using familywise corrected *P*-FDR < 0.05 connection- and cluster-level threshold.^74^ To directly compare the effects of the association between the FRS and functional connectivity between AD and FTLD, we applied a subsampling framework^75,76^ to the subset of connections that were statistically significant in either AD or FTLD models (*n* = 15). We conducted 1000 iterations of stratified random subsampling for each connection without replacement. In each iteration, we fitted an ordinary least squares (OLS) regression model predicting functioning connectivity from FRS scores, while adjusting for age, sex, and scanner type. The *t*-value associated with the RS coefficient was extracted from each model and stored, yielding empirical distributions of *t*-values for AD and FTLD, representing the variability of the FRS-functional connectivity associations across groups. Next, we performed independent sample *t*-tests to compare the distribution of *t*-values, allowing us to test the differential impact of FRS on functional connectivity between AD and FTLD.

### 2.5 Data availability statement

Anonymized data that support the study findings are drawn from the BrainLat project,^41^ a large open access multimodal neuroimaging database that can be found here: https://www.synapse.org/Synapse:syn51549340/wiki/624187.^77^

## 3. Results

### 3.1 Demographics, cognitive performance and cardiovascular risk

No significant differences were observed between age and sex between FTLD and CN or AD and CN following matching (*Table 1*, Supplementary material online, *Tables S1* and *S2* for subtype analyses). In both tandems (FTLD-CN, and AD-CN), patients had worse cognitive scores than the controls (both *p*’s < 0.001). FTLD and AD were in mild-to-moderate disease stages, on average. Cardiovascular risk scores did not differ between patients with dementia syndromes and CN tandems (both *p*’s > 05).

**Table 1:** Demographic and neuropsychological assessment between patients and control tandems

	CN	FTLD	Statistic	*P*
	(*n* = 304)	(*n* = 304)		
Age	64.77 ± 8.60	65.44 ± 7.86	−0.99	0.320
Sex (M:F)	166:138	166:138	0.00	1.000
Education	14.56 ± 5.46	14.21 ± 4.15	0.85	0.395
MMSE	28.14 ± 3.00	21.52 ± 6.47	15.97	<0.001
CDR-FTLD SoB	-	8.64 ± 3.97	-	-
FRS	13.67 ± 3.99	13.80 ± 3.84	−0.40	0.687

	CN	AD	Statistic	*P*
	(n = 432)	(n = 429)		
Age	67.68 ± 7.25	68.33 ± 7.54	1.29	0.195
Sex (M:F)	145:287	151:278	0.19	0.665
Education	13.27 ± 5.92	12.92 ± 4.92	−0.96	0.335
MMSE	27.50 ± 3.36	19.92 ± 4.92	−23.40	<0.001
CDR SoB	-	5.82 ± 2.98	-	-
FRS	14.54 ± 3.90	15.01 ± 4.14	1.70	0.090

AD, Alzheimer’s disease; CDR-SoB, Clinical Dementia Rating Sum of Boxes; CN, controls; FRS, Framingham’s Risk Score; FTLD, frontotemporal lobar degeneration; MMSE, Mini Mental State Examination

### 3.2 Increased cardiovascular risk is associated with reduced structural integrity of the AIN in dementia

Expected patterns of atrophy were observed in each clinical syndrome compared with CNs (see Supplementary material online, *Figures S1* and *S2*; *Tables S6*–*S16*).

**Figure 2:**
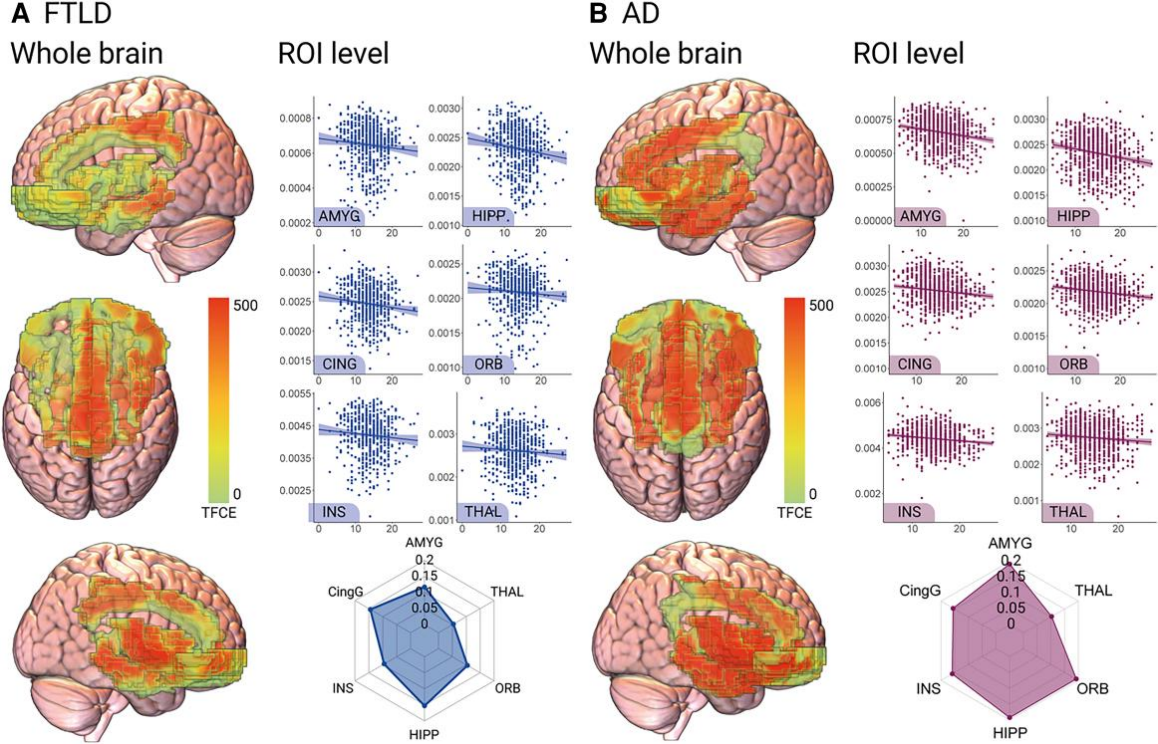
Brain volume of the allostatic interoceptive network and cardiovascular risk in dementia. Reduced structural integrity associated with increased cardiovascular risk in *A*) FTLD syndromes and *B*) in AD syndromes. Whole brain plots display VBM results with TFCE values shown within predefined regions, with FDR *P* < 0.05. Scatterplots show GM volumes within predefined regions associated with cardiovascular risk scores. Spider plots display Pearson *r*-values for correlations between each ROI and FRS score. AD, Alzheimer’s disease; AMYG, Amygdala; CING, Cingulate; FTLD, Frontotemporal Lobar Degeneration; HIPP, Hippocampus; INS, Insula; ORB, Orbitofrontal cortex; THAL, Thalamus.

In FTLD, higher cardiovascular risk was associated with reduced structural integrity of the bilateral insula, thalamus, anterior cingulate cortex, and paracingulate cortex, and right amygdala, hippocampus, parahippocampus, temporal pole, and superior temporal gyrus. (*Figure 2A*, Supplementary material online, *Table S16*). Similar results were observed when controlling for FTD subtype (see Supplementary material online, *Table S17*).

In AD, higher cardiovascular risk scores were associated with reduced structural integrity of the bilateral amygdala, hippocampus, parahippocampal gyrus, superior temporal gyrus, temporal pole, insula, thalamus, anterior cingulate cortex, and paracingulate cortex (*Figure 2B*, Supplementary material online, *Table S18*). Similar results were observed when controlling for AD subtype (see Supplementary material online, *Table S19*).

**Figure 3:**
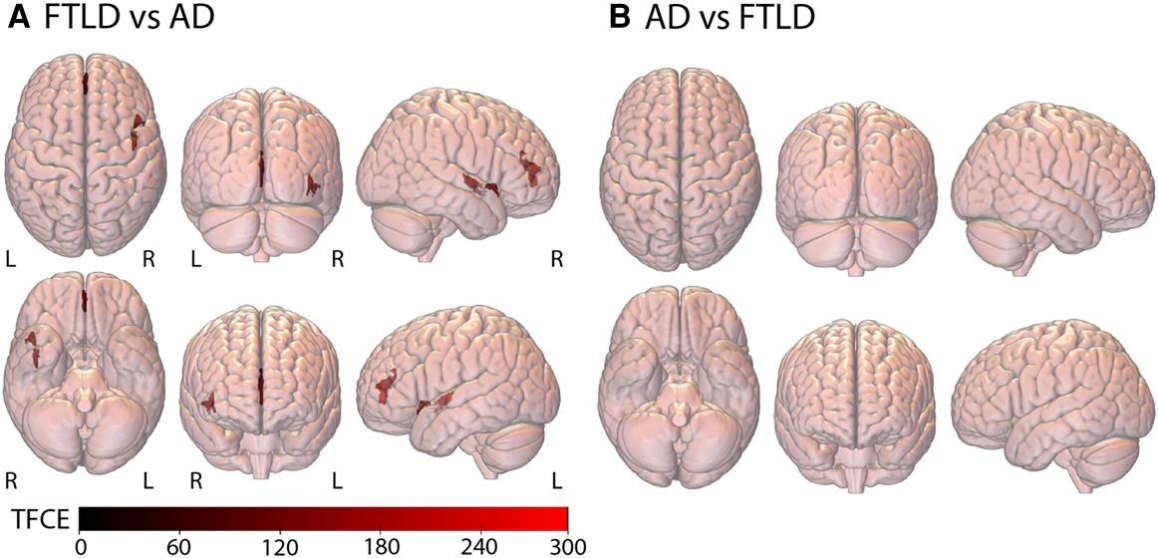
Reduced grey matter integrity associated with increased cardiovascular risk in *A*) FTLD vs. AD, and *B*) AD vs. FTLD. Whole brain plots display VBM results with TFCE values shown in predefined regions, with FDR *P* < 0.05 using a 50 contiguous voxel threshold.

Direct comparisons between AD and FTLD after accounting for age and sex revealed that increased cardiovascular risk was associated with reduced grey matter integrity in key AIN regions (e.g. bilateral ACC, right insula) in FTLD compared with AD (*Figure 3A*, Supplementary material online, *Table S20*). No clusters were observed where increased cardiovascular risk was associated with reduced grey matter integrity in AD compared with FTLD (*Figure 3B*).

### 3.3 Increased cardiovascular risk is associated with reduced AIN connectivity in dementia

**Figure 4:**
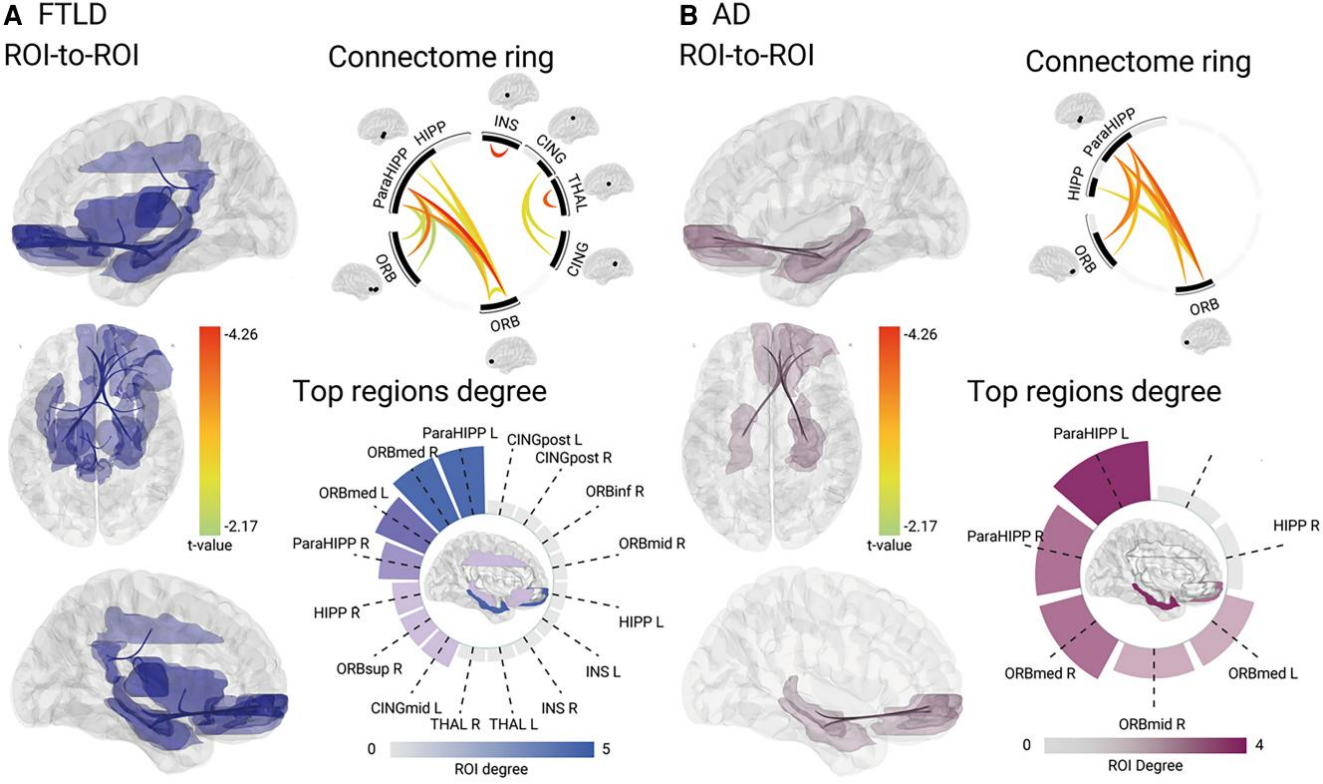
Allostatic interoceptive network functionality and cardiovascular risk in dementia. Reduced functional connectivity associated with increased cardiovascular risk in *A*) FTLD syndromes; and *B*) AD syndromes. In each panel, ROI-to-ROI connectivity maps are shown. Connectome rings show the strength of connectivity between each ROI, with colour bars representing the connectivity strength using *t*-values. Radial plots show the number of connections of each ROI to different regions, with colour bars representing the maximum number of ROI connections. CINGmid, Middle cingulate cortex; CINGpost, Posterior cingulate cortex; HIPP, Hippocampus; INS, Insula; ORBinf, Orbitofrontal cortex (inferior); ORBmed, Orbitofrontal cortex (medial); ORBmid, Orbitofrontal cortex (middle); ORBsup, Orbitofrontal cortex (superior); ParaHIPP, Parahippocampal gyrus; THAL, Thalamus.

In FTLD, higher cardiovascular risk was associated with reduced resting-state functional connectivity in six clusters in FTLD (*Figure 4A*, Supplementary material online, *Table S20*), including the bilateral insula (cluster 1), bilateral thalamus (cluster 2), bilateral parahippocampal gyrus, orbitofrontal cortex (medial part), and bilateral hippocampus (cluster 3), bilateral parahippocampal gyrus and orbitofrontal cortex (inferior, superior, and middle parts) (cluster 4), bilateral middle cingulate cortex and bilateral posterior cingulate cortex (cluster 5) and bilateral orbitofrontal cortex (medial part) (cluster 6).

In AD, higher cardiovascular risk was associated with reduced resting-state functional connectivity in two clusters, involving bilateral parahippocampal gyrus, orbitofrontal cortex (medial part) and hippocampus (cluster 1), and the bilateral parahippocampal gyrus and orbitofrontal cortex (middle and superior) (cluster 2) (*Figure 4B*, Supplementary material online, *Table S21*).

Finally, we compared functional connectivity in FTLD and AD directly, based on the significant connections observed in each group separate (*n* = 15 connections). Here, a stronger effect was observed for higher cardiovascular risk in FTLD than in AD for 14/15 of the connections (*Table 2*), including reduced connectivity between the bilateral orbitofrontal cortex, hippocampus, parahippocampus, insula, and the left middle and posterior cingulate cortex. The reverse pattern was observed for one connection, where a stronger effect for higher cardiovascular risk was observed in AD than in FTLD in the right orbitofrontal cortex and left parahippocampal gyrus. These results largely mirror the pattern of results observed in the groups separately.

**Table 2:** Functional connectivity comparisons between FTLD and AD using 1000 subsampling models per group

Connection	FTLD M ± SD	AD M ± SD	*t*	*P*
Frontal Med Orb R & Hippocampus L	−1.64 ± 0.80	−0.18 ± 0.82	−40.48	<0.0.000001
Frontal Med Orb L & Hippocampus L	−1.34 ± 0.80	0.08 ± 0.81	−39.35	<0.0.000001
Cingulum Mid L & Cingulum Post L	−2.09 ± 0.76	−0.64 ± 0.89	−39.09	<0.0.000001
Thalamus L & Thalamus R	−1.48 ± 0.79	−0.41 ± 0.89	−28.29	<0.0.000001
Frontal Med Orb R & Parahippocampal L	−1.99 ± 0.75	−1.01 ± 0.85	−27.15	<0.0.000001
Frontal Sup Orb R & Parahippocampal L	−1.42 ± 0.69	−0.68 ± 0.79	−22.51	<0.0.000001
Cingulum Mid L & Cingulum Post R	−1.50 ± 0.77	−0.69 ± 0.84	−22.46	<0.0.000001
Frontal Med Orb L & Parahippocampal L	−1.67 ± 0.84	−0.95 ± 0.84	−19.16	<0.0.000001
Insula L & Insula R	−1.34 ± 0.75	−0.64 ± 0.98	−17.95	<0.0.000001
Frontal Med Orb L & Hippocampus R	−1.12 ± 0.77	−0.50 ± 0.85	−17.18	<0.0.000001
Frontal Med Orb R & Parahippocampal R	−1.56 ± 0.75	−0.93 ± 0.91	−16.99	<0.0.000001
Frontal Sup Orb R & Parahippocampal R	−0.64 ± 0.70	−0.11 ± 0.73	−16.57	<0.0.000001
Frontal Inf Orb R & Parahippocampal L	−0.87 ± 0.80	−0.41 ± 0.85	−12.38	<0.0.000001
Frontal Med Orb R & Hippocampus R	−1.00 ± 0.76	−0.83 ± 0.82	−4.77	0.000002
Frontal Mid Orb R & Parahippocampal L	−0.72 ± 0.71	−1.00 ± 0.80	8.12	<0.0.000001

Subsampling for each group *N* = 1000, degrees of freedom (df) for each comparison = 1000.

AD, Alzheimer’s Disease; FTLD, Frontotemporal Dementia; M, Mean; SD, Standard deviation.

## 4. Discussion

Our study provides the first evidence that cardiovascular risk factors are associated with substantial structural and functional features within the AIN in both FTLD and AD. While higher cardiovascular risk correlated with reduced structural integrity in similar brain regions of the AIN in both FTLD and AD, differences were observed in functional connectivity metrics, highlighting disease-specific network vulnerabilities. In FTLD, widespread reduced functional connectivity associated with greater cardiovascular risk was observed in the bilateral insula, cingulate cortex, orbitofrontal cortex, thalamus, and hippocampus, mirroring structural correlates. In AD, reduced connectivity was circumscribed within the hippocampus, parahippocampus, and orbitofrontal cortices. Further, direct comparisons between disease phenotypes revealed a stronger effect associated with cardiovascular risk in FTLD than in AD in both structural and functional analyses, when accounting for demographic differences. Our findings highlight similar structural burdens associated with cardiovascular risk, but the observed disease-specific functional alterations suggest that distinct pathways and network vulnerabilities are involved in cardiovascular risk in FTLD and AD. In the following paragraphs, we will consider the theoretical and clinical implications of this work, as well as the relevance for public policy and health initiatives to promote dementia prevention and improve dementia care.

Both structural and functional alterations within the AIN were associated with cardiovascular risk factors in FTLD and AD, which remained significant when considering disease subtypes within each syndrome. In FTLD, widespread structural and functional connectivity alterations were associated with cardiovascular risk in the AIN. This finding fits with emerging evidence of multimodal allostatic-interoceptive disruptions spanning behavioral, peripheral, and neural measures occurring within this syndrome, particularly within bvFTD.^13,18–25,27^ Taken together, this evidence suggests that allostatic overload likely influences and exacerbates disease mechanisms in FTLD syndromes, based on observed damage within the AIN.^6,7,18^ Somewhat surprisingly, in AD increased cardiovascular risk was also associated with widespread structural volume reductions in the AIN, whereas functional neuroimaging analyses in AD revealed a more targeted pattern of connectivity disruptions in select AIN regions, such as the hippocampus, parahippocampus, and orbitofrontal cortex. Prior studies have reported altered allostatic markers in AD,^26,28–31^ noting that cardiometabolic burden may have a more substantial impact on AD risk than genetic factors.^5^ Therefore, a plausible mechanism underlying this disruption in AD is impaired insulin signaling in the brain.^29^ Impaired insulin signaling has a bidirectional relationship with allostatic load and has been proposed to exacerbate AD pathophysiology,^29^ particularly within the hippocampus^78^ and in more vulnerable populations^29^ similar to our patient cohort. In sum, our findings support the predictive coding theory of allostatic interoception,^6–11^ by showing that cardiovascular stress potentially disrupts the brain’s adaptive mechanisms likely via prolonged allostatic overload in both FTLD and AD. Further, reduced AIN connectivity and structural atrophy in FTLD and AD suggest that cardiovascular risks may hasten neurodegeneration by impairing interoceptive and emotional processing pathways^6,7,13,18^

The current work has several strengths. First, cardiovascular health was associated with brain structure and function in two distinct dementia syndromes within the AIN, even in the absence of major cardiovascular compromise (i.e. no differences were observed in cardiovascular risk between controls and patients). This study extends on previous literature focusing on cardiovascular risk and hippocampal volume in healthy aging^32,33^ and highlights how cardiovascular burden manifests in disruptions in AIN structure and function in dementia. Further, our results support synergistic embodied health approaches that consider whole-body health in brain health.^3,4^ Routine cardiovascular assessments in clinical settings could be a valuable and actionable addition to dementia care and prognosis.^79^ Indeed, mid-life cardiovascular risk factors are among the strongest predictors of later life dementia.^1^ Additionally, we assessed a large cohort including both Latin American and US participants using the FRS, a well-validated, widely used, and easily implementable measure of cardiovascular risk.^40^ The FRS is particularly suitable for studies in Latin American populations where harmonization across diverse sites is essential. Its inclusion of age, blood pressure, cholesterol, smoking, and diabetes aligns closely with known contributors to allostatic load and interoceptive dysfunction.^14–17^ Moreover, FRS has been applied across multiple global and LMIC settings,^56–59^ facilitating comparability and enabling integration with existing epidemiological data. This makes it a pragmatic and theoretically grounded tool for examining how cardiovascular burden affects the allostatic-interoceptive brain network in underserved populations. This cross-cultural approach provides much needed insights into underrepresented populations in dementia research. Recent evidence has highlighted greater structural inequalities as well as accelerated brain aging in dementia in Latin America compared with other parts of the world,^68,80,81^ combined with increased cardiovascular risk in this region.^37–39^ Although speculative, this work suggests that increased prevalence of cardiovascular risk factors and increased prevalence of dementia in Latin America^37–39,82^ may be driven by allostatic overload and may converge within the AIN. Finally, our multimodal neuroimaging approach allowed for a thorough examination of grey matter volume and functional connectivity of the AIN in dementia syndromes, offering a novel perspective on whole-body health in neurodegeneration.

The current study has some limitations that call for further research. First, our cross-sectional design limits any direct causal interpretations between cardiovascular risk factors and neurodegenerative processes. Longitudinal studies are necessary to confirm causality between cardiovascular risk and structural and functional disruptions within the AIN. In addition, whether addressing cardiovascular risk factors in routine clinical practice in people with dementia has an impact on dementia prognosis warrants attention. Next, the cardiovascular measure we used, namely the FRS, was limited to non-laboratory measures as laboratory measures were not available across research centers. Although previous evidence suggest non-laboratory measures these are comparable with laboratory measures in measuring cardiovascular risk,^56–59^ other biomarkers associated with cardiometabolic risk and/or allostatic load (e.g. cholesterol, cortisol), and other relevant physical measures such as waist-to-hip ratio, or key lifestyle factors, such as physical inactivity, nutrition, life-time cigarette burden, and alcohol consumption measures were not measured. Future research is needed to determine how these factors may influence neurodegeneration, potentially via epigenetic mechanisms.^83–85^ In addition, demographic differences precluded comparisons between AD and FTLD, and a small portion of AD cases were excluded due to older age. Therefore, it is currently unknown whether differences in cardiovascular risk profiles between these dementia syndromes exist, as well as during different stages of dementia, and warrants further consideration. Finally, we did not measure genetic mutations in this study. Over two-thirds FTLD cases are considered to be ‘sporadic’, with no currently known genetic cause^86^ and research suggests that cardiovascular risk factors predict the likelihood of AD beyond genetic factors alone.^5^ A recent study also reported greater prevalence of cardiovascular disease in sporadic than genetic FTD.^87^ The contribution of cardiovascular risk factors could be more pronounced in ‘sporadic’ FTLD and AD due to prolonged allostatic overload.^6,7,13,18^ Future research comparing genetic vs. sporadic cohorts will be useful to shed light on this topic.

In conclusion, the current study evidenced substantial associations between cardiovascular health and AIN integrity in dementia. This work aligns with predictive coding theories,^6–13^ highlighting the role of cumulative cardiovascular stress on allostatic interoception networks vulnerable to dementia pathology. The management of cardiovascular risk factors could represent a key intervention strategy for dementia syndromes, potentially by reducing allostatic load on the AIN. Future work is needed to uncover longitudinal effects of cardiovascular risk on dementia and to determine if cardiovascular risk factors exacerbate neurodegenerative processes, together with clinical consideration of cardiovascular health in dementia diagnosis to minimize disease burden and improve patient outcomes.

## Supplementary Material

cvaf185_Supplementary_Data

## Data Availability

The data supporting the findings of this ReDLat study contain sensitive clinical information and cannot be made publicly available due to ethical and legal restrictions across participating Latin American sites. De-identified data and associated metadata are available upon reasonable request to the ReDLat Data Access Committee (contact: redlat.coordinacion@gmail.com) for researchers who meet the criteria for access to confidential data. All shared data will comply with institutional and national regulations, and accompanying documentation and analysis code are available to enable reproducibility.
