# Supplementary material for: Cardiovascular risk factors and the allostatic interoceptive network in dementia
Source: Cardiovasc Res. 2025 Oct 10;121(14):2222–32. doi: 10.1093/cvr/cvaf185 (PMC12638725; doi:10.1093/cvr/cvaf185)
Supplement: cvaf185_Supplementary_Data [file cvaf185_supplementary_data.docx]

**Supplementary materials**

**Supplementary methods**

**Demographics, cognitive assessment, and cardiovascular risk in FTLD and AD subtypes**

Demographics, cognitive assessment, and cardiovascular risk was compared between FTD subtypes and controls (Supplementary Table 1) and AD subtypes and controls (Supplementary Table 2).

**FTD subtypes**

In brief, no significant differences were found for age or sex in FTD subtypes and controls. FTD mixed (corticobasal syndrome, progressive supranuclear palsy and FTD with motor neuron disease) had fewer years of education compared to bvFTD and controls. All FTD subtypes showed worse cognitive performance than controls, and nfvPPA had lower cognitive scores than bvFTD. bvFTD and svPPA had a more severe dementia stage than nfvPPA, and bvFTD had a more severe disease stage than FTD-mixed. Cardiovascular risk scores were not significantly different between FTD subtypes and controls.

**AD subtypes**

In AD subtypes, PCA were significantly younger than ADa. Further, fv-AD and lvPPA had the reversed pattern of expected frequencies for sex. ADa had fewer years of education compared to lvPPA. All AD subtypes showed worse cognitive performance than controls. All AD-subtype patients were at a mild-to-moderate disease stage on average. Notable differences in disease stages were that PCA patients had a more severe disease stage than lvPPA. Finally, PCA patients had a lower cardiovascular risk score compared to ADa patients.

**Supplementary Table 1.** Demographics in FTD subtypes compared to controls.

|  | **CN** | **bvFTD** | **nfvPPA** | **svPPA** | **FTD mixed ^a^** | **Statistic** | ***p*** | **Post hoc** |
| --- | --- | --- | --- | --- | --- | --- | --- | --- |
|  | (n = 304) | (n = 189) | ( n = 38) | (n = 52) | (n = 25) |  |  |  |
| Age | 64.78 ± 8.60 | 64.49 ± 7.99 | 67.5 ± 7.43 | 66.27 ±7.54 | 67.76 ± 7.25 | 2.11 | 0.078 | ns |
| Sex (M:F) | 166:138 | 113:76 | 16:22 | 23:29 | 14:11 | 11.48 | 0.175 | ns |
| Education | 14.56 ± 5.46 | 14.68 ± 4.14 | 13.68 ± 4.83 | 14.31 ± 4.59 | 11.32 ± 4.54 | 2.87 | 0.022 | FTD mixed < bvFTD & CN |
| MMSE | 28.14 ± 3.00 | 22.11 ± 6.43 | 19.46 ± 8.97 | 20.12 ± 4.91 | 22.96 ± 4.24 | 69.71 | <.001 | All < CN; nfvPPA < bvFTD |
| CDR | - | 9.38 ± 3.79 | 6.17 ± 4.97 | 8.62 ± 2.98 | 6.96 ± 3.51 | 9.28 | <.001 | nfvPPA < svPPA & bvFTD  FTD mixed < bvFTD |
| FRS | 13.67 ± 3.99 | 13.66 ± 3.95 | 14.29 ± 3.97 | 13.71 ± 3.27 | 14.36 ± 4.07 | 0.39 | 0.818 | ns |

Note. Post hoc tests were conducted using Tukey HSD, FWE *p* <.05; ^a^ FTD mixed = 9 CBS, 12 PSP, 2 FTD-MND. Abbreviations: bvFTD = behavioral-variant frontotemporal dementia; nfvPPA = nonfluent-variant of primary progressive aphasia; svPPA = semantic-variant primary progressive aphasia; FTD = frontotemporal dementia; CBS = Corticobasal syndrome; CN = controls; PSP = Progressive supranuclear palsy’ FTD-MND = FTD with motor neuron disease; ns = not significant.

**Supplementary Table 2.** Demographics in AD subtypes compared to controls.

|  | **CN** | **ADa** | **Atypical AD** | | | **Statistic** | ***p*** | **Post hoc** |
| --- | --- | --- | --- | --- | --- | --- | --- | --- |
|  |  |  | **lvPPA** | **PCA** | **fv-AD** |  |  |  |
|  | (n = 432) | (n = 375) | (n = 24) | (n = 13) | (n =17) |  |  |  |
| Age | 67.68 ± 7.25 | 69.07 ± 7.42 | 66.08 ± 6.59 | 62.54 ± 7.24 | 65.41 ± 8.28 | 4.88 | <.001 | PCA < ADa |
| Sex (M:F) | 113:241 | 145:287 | 14:10 | 5:8 | 11:6 | 14.33 | 0.014 | fv-AD & lvPPA only ^a^ |
| Education | 13.27 ± 5.92 | 12.47 ± 4.93 | 15.75 ± 4.11 | 14.69 ± 4.55 | 14.88 ± 3.77 | 3.09 | 0.009 | ADa < lvPPA |
| MMSE | 27.50 ± 3.36 | 20.50 ± 5.18 | 18.33 ± 8.23 | 16.83 ± 4.55 | 18.35 ± 6.53 | 123.39 | <.001 | All patients < CN |
| CDR | - | 5.79 ± 2.81 | 5.0 ± 2.73 | 7.77 ± 3.15 | 5.32 ± 1.86 | 2.65 | 0.032 | lvPPA < PCA |
| FRS | 14.54 ± 3.90 | 15.23 ± 4.18 | 14.29 ± 3.21 | 11.77 ± 3.15 | 14.24 ± 3.60 | 2.80 | 0.016 | PCA < ADa |

Note. Post hoc tests were conducted using Tukey HSD, FWE *p* < .05; ^a^ For fv-AD & lvPPA, the actual sex frequencies were reversed compared to the expected sex frequencies. Abbreviations: ADa = Alzheimer’s Disease amnestic; lvPPA = logopenic-variant of primary progressive aphasia; PCA = posterior cortical atrophy; fv-AD = frontal-variant of Alzheimer’s disease.

**Measures**

**Framingham’s Risk Score calculation**

The Framingham’s Risk Score (1) was calculated based on non-laboratory measures. Scoring is presented in Supplementary Table 3. Body mass index (BMI) was measured as a ratio between weight (kg) and height (cm) squared. Hypertension status (based on clinical reports and medication use) was taken into account when scoring systolic blood pressure. Smoking status related to the participant’s self-report of smoking. Diabetes status was based on clinical reports and medication use.

Supplementary Table 3. Framingham’s risk score calculations based on non-laboratory measures in females and males.

| **Points** | **Age** | **BMI** | **SBP**  **(not treated)** | **SBP (treated)** | **Smoker** | **Diabetes** |
| --- | --- | --- | --- | --- | --- | --- |
| Women |  |  |  |  |  |  |
| -3 |  |  | <120 |  |  |  |
| -1 |  |  |  | <120 |  |  |
| 0 | 30-34 | <25 | 120-129 |  | No | No |
| 1 |  | 25-29.9 | 130-139 |  |  |  |
| 2 | 35-39 | >=30 |  | 120-129 |  |  |
| 3 |  |  | 140-149 | 130-139 |  |  |
| 4 |  |  | 150-159 |  | Yes |  |
| 5 | 40-44 |  | 160+ | 140-149 |  | Yes |
| 6 | 45-49 |  |  | 150-159 |  |  |
| 8 | 50-54 |  |  | 160+ |  |  |
| 10 | 55-59 |  |  |  |  |  |
| 11 | 60-64 |  |  |  |  |  |
| 12 | 65-69 |  |  |  |  |  |
| 14 | 70-74 |  |  |  |  |  |
| 15 | 75+ |  |  |  |  |  |
| **Men** |  |  |  |  |  |  |
| -2 |  |  |  |  |  |  |
| 0 | 30-34 | <25 | 120-129 | <120 | No | No |
| 1 |  | 25-29.9 | 130-139 |  |  |  |
| 2 | 35-39 | >=30 | 140-149 | 120-129 |  |  |
| 3 |  |  | 160+ | 130-139 |  | Yes |
| 4 |  |  |  | 140-159 | Yes |  |
| 5 | 40-44 |  |  | 160+ |  |  |
| 7 | 45-49 |  |  |  |  |  |
| 8 | 50-54 |  |  |  |  |  |
| 10 | 55-59 |  |  |  |  |  |
| 11 | 60-64 |  |  |  |  |  |
| 13 | 65-69 |  |  |  |  |  |
| 14 | 70-74 |  |  |  |  |  |
| 15 | 75+ |  |  |  |  |  |

Note. Abbreviations: BMI = Body Mass Index; SBP = Systolic Blood Pressure

**Resting state functional connectivity analysis**

**Preprocessing**. Functional data were smoothed using spatial convolution with a Gaussian kernel of 6 mm full width half maximum (FWHM).

**Denoising.** In addition, functional data were denoised using a standard denoising pipeline [(2)](https://web.endnote.com/citations/eyJkaXNwbGF5VGV4dCI6IigxKSIsImNpdGF0aW9ucyI6W3siZ3VpZCI6IjVmYzcwNzAwLWYxNGYtNDI5Mi1hMTBmLTdlMjQ4ZjYxMTgxOCIsImJpYmxpb0NvbnRlbnQiOlt7InllYXIiOiIyMDIwIiwiZ3VpZCI6IjVmYzcwNzAwLWYxNGYtNDI5Mi1hMTBmLTdlMjQ4ZjYxMTgxOCIsInJlY29yZFN0YXR1cyI6ImFjdGl2ZSIsImdyb3VwR3VpZHMiOltdLCJ0aXRsZSI6IkhhbmRib29rIG9mIGZ1bmN0aW9uYWwgY29ubmVjdGl2aXR5IG1hZ25ldGljIHJlc29uYW5jZSBpbWFnaW5nIG1ldGhvZHMgaW4gQ09OTiIsInJlZmVyZW5jZVR5cGUiOiI2IiwicHVibGlzaGVyIjoiSGlsYmVydCBQcmVzcyIsImlzYm4iOiIwNTc4NjQ0MDAyIiwiYXV0aG9ycyI6WyJOaWV0by1DYXN0YW5vbiwgQWxmb25zbyJdfV0sImdyb3VwR3VpZHMiOltdfV19) including the regression of potential confounding effects characterized by white matter timeseries (5 CompCor noise components), CSF timeseries (5 CompCor noise components), motion parameters and their first order derivatives (12 factors)[(3)](https://web.endnote.com/citations/eyJkaXNwbGF5VGV4dCI6IigyKSIsImNpdGF0aW9ucyI6W3siZ3JvdXBHdWlkcyI6W10sImJpYmxpb0NvbnRlbnQiOlt7Im51bWJlciI6IjMiLCJ2b2x1bWUiOiIzNSIsInNlY29uZGFyeVRpdGxlIjoiTWFnbmV0aWMgcmVzb25hbmNlIGluIG1lZGljaW5lIiwiZ3VpZCI6IjIxNGYyZjFlLTgzNzktNDRkYS05MGQwLWJmYjZhMDg2Njg2NiIsInllYXIiOiIxOTk2IiwiYXV0aG9ycyI6WyJGcmlzdG9uLCBLYXJsIEoiLCJXaWxsaWFtcywgU3RldmVuIiwiSG93YXJkLCBSb2JlcnQiLCJGcmFja293aWFrLCBSaWNoYXJkIFNKIiwiVHVybmVyLCBSb2JlcnQiXSwidGl0bGUiOiJNb3ZlbWVudOKAkHJlbGF0ZWQgZWZmZWN0cyBpbiBmTVJJIHRpbWXigJBzZXJpZXMiLCJpc2JuIjoiMDc0MC0zMTk0IiwiZ3JvdXBHdWlkcyI6W10sInJlZmVyZW5jZVR5cGUiOiIxNyIsInBhZ2VzIjoiMzQ2LTM1NSIsInJlY29yZFN0YXR1cyI6ImFjdGl2ZSJ9XSwiZ3VpZCI6IjIxNGYyZjFlLTgzNzktNDRkYS05MGQwLWJmYjZhMDg2Njg2NiJ9XX0%3D), outlier scans (below 526 factors)[(4)](https://web.endnote.com/citations/eyJkaXNwbGF5VGV4dCI6IigzKSIsImNpdGF0aW9ucyI6W3siZ3VpZCI6ImE5MjgyODFmLTcyOTItNDFiZS1iZGQzLTZiNzQxNDg3OGQyMiIsImJpYmxpb0NvbnRlbnQiOlt7InNlY29uZGFyeVRpdGxlIjoiTmV1cm9pbWFnZSIsInBhZ2VzIjoiMzIwLTM0MSIsInJlY29yZFN0YXR1cyI6ImFjdGl2ZSIsImdyb3VwR3VpZHMiOltdLCJ5ZWFyIjoiMjAxNCIsInJlZmVyZW5jZVR5cGUiOiIxNyIsImd1aWQiOiJhOTI4MjgxZi03MjkyLTQxYmUtYmRkMy02Yjc0MTQ4NzhkMjIiLCJhdXRob3JzIjpbIlBvd2VyLCBKb25hdGhhbiBEIiwiTWl0cmEsIEFuaXNoIiwiTGF1bWFubiwgVGltb3RoeSBPIiwiU255ZGVyLCBBYnJhaGFtIFoiLCJTY2hsYWdnYXIsIEJyYWRsZXkgTCIsIlBldGVyc2VuLCBTdGV2ZW4gRSJdLCJpc2JuIjoiMTA1My04MTE5IiwidGl0bGUiOiJNZXRob2RzIHRvIGRldGVjdCwgY2hhcmFjdGVyaXplLCBhbmQgcmVtb3ZlIG1vdGlvbiBhcnRpZmFjdCBpbiByZXN0aW5nIHN0YXRlIGZNUkkiLCJ2b2x1bWUiOiI4NCJ9XSwiZ3JvdXBHdWlkcyI6W119XX0%3D), session and task effects and their first order derivatives (2 factors), and linear trends (2 factors) within each functional run, followed by bandpass frequency filtering of the BOLD time series [(5)](https://web.endnote.com/citations/eyJkaXNwbGF5VGV4dCI6Iig0KSIsImNpdGF0aW9ucyI6W3siZ3VpZCI6ImI3YTk4OTFiLTUxYTUtNDVlMi1hYWRlLWIxMGM0MTY0MzI0YiIsImJpYmxpb0NvbnRlbnQiOlt7InNlY29uZGFyeVRpdGxlIjoiTmV1cm9pbWFnZSIsImF1dGhvcnMiOlsiSGFsbHF1aXN0LCBNaWNoYWVsIE4iLCJId2FuZywgS2FpIiwiTHVuYSwgQmVhdHJpeiJdLCJ0aXRsZSI6IlRoZSBudWlzYW5jZSBvZiBudWlzYW5jZSByZWdyZXNzaW9uOiBzcGVjdHJhbCBtaXNzcGVjaWZpY2F0aW9uIGluIGEgY29tbW9uIGFwcHJvYWNoIHRvIHJlc3Rpbmctc3RhdGUgZk1SSSBwcmVwcm9jZXNzaW5nIHJlaW50cm9kdWNlcyBub2lzZSBhbmQgb2JzY3VyZXMgZnVuY3Rpb25hbCBjb25uZWN0aXZpdHkiLCJpc2JuIjoiMTA1My04MTE5Iiwidm9sdW1lIjoiODIiLCJwYWdlcyI6IjIwOC0yMjUiLCJ5ZWFyIjoiMjAxMyIsImdyb3VwR3VpZHMiOltdLCJyZWNvcmRTdGF0dXMiOiJhY3RpdmUiLCJndWlkIjoiYjdhOTg5MWItNTFhNS00NWUyLWFhZGUtYjEwYzQxNjQzMjRiIiwicmVmZXJlbmNlVHlwZSI6IjE3In1dLCJncm91cEd1aWRzIjpbXX1dfQ%3D%3D) between 0.008 Hz and 0.09 Hz. CompCor[(6, 7)](https://web.endnote.com/citations/eyJkaXNwbGF5VGV4dCI6Iig1LCA2KSIsImNpdGF0aW9ucyI6W3siZ3VpZCI6ImQ2OGQyZGZhLTI2NTEtNDQ1OS1iNTI4LTJkMzQ0ZDgyNThlNCIsImJpYmxpb0NvbnRlbnQiOlt7InBhZ2VzIjoiOTAtMTAxIiwic2Vjb25kYXJ5VGl0bGUiOiJOZXVyb2ltYWdlIiwicmVmZXJlbmNlVHlwZSI6IjE3IiwiYXV0aG9ycyI6WyJCZWh6YWRpLCBZYXNoYXIiLCJSZXN0b20sIEtoYWxlZCIsIkxpYXUsIEpveSIsIkxpdSwgVGhvbWFzIFQiXSwiZ3JvdXBHdWlkcyI6W10sInRpdGxlIjoiQSBjb21wb25lbnQgYmFzZWQgbm9pc2UgY29ycmVjdGlvbiBtZXRob2QgKENvbXBDb3IpIGZvciBCT0xEIGFuZCBwZXJmdXNpb24gYmFzZWQgZk1SSSIsImlzYm4iOiIxMDUzLTgxMTkiLCJyZWNvcmRTdGF0dXMiOiJhY3RpdmUiLCJ2b2x1bWUiOiIzNyIsInllYXIiOiIyMDA3IiwiZ3VpZCI6ImQ2OGQyZGZhLTI2NTEtNDQ1OS1iNTI4LTJkMzQ0ZDgyNThlNCIsIm51bWJlciI6IjEifV0sImdyb3VwR3VpZHMiOltdfSx7Imdyb3VwR3VpZHMiOltdLCJndWlkIjoiZDIwNmU4NWQtNDdmMi00MDFjLWJiMTktMDkyNjdiZGI2ZTQ0IiwiYmlibGlvQ29udGVudCI6W3siZ3VpZCI6ImQyMDZlODVkLTQ3ZjItNDAxYy1iYjE5LTA5MjY3YmRiNmU0NCIsImdyb3VwR3VpZHMiOltdLCJ0aXRsZSI6IkFudGljb3JyZWxhdGlvbnMgaW4gcmVzdGluZyBzdGF0ZSBuZXR3b3JrcyB3aXRob3V0IGdsb2JhbCBzaWduYWwgcmVncmVzc2lvbiIsImlzYm4iOiIxMDUzLTgxMTkiLCJ2b2x1bWUiOiI1OSIsInJlY29yZFN0YXR1cyI6ImFjdGl2ZSIsInllYXIiOiIyMDEyIiwicmVmZXJlbmNlVHlwZSI6IjE3IiwibnVtYmVyIjoiMiIsInNlY29uZGFyeVRpdGxlIjoiTmV1cm9pbWFnZSIsInBhZ2VzIjoiMTQyMC0xNDI4IiwiYXV0aG9ycyI6WyJDaGFpLCBYaWFvcWlhbiBKIiwiQ2FzdGHDscOzbiwgQWxmb25zbyBOaWV0byIsIsOWbmfDvHIsIERvc3QiLCJXaGl0ZmllbGQtR2FicmllbGksIFN1c2FuIl19XX1dfQ%3D%3D) noise components within white matter and CSF were estimated by computing the average BOLD signal as well as the largest principal components orthogonal to the BOLD average, motion parameters, and outlier scans within each subject's eroded segmentation masks. From the number of noise terms included in this denoising strategy, the effective degrees of freedom of the BOLD signal after denoising were estimated to range from 0 to 107.7 (average 52.3) across all subjects [(8)](https://web.endnote.com/citations/eyJkaXNwbGF5VGV4dCI6Iig3KSIsImNpdGF0aW9ucyI6W3siYmlibGlvQ29udGVudCI6W3siZ3JvdXBHdWlkcyI6W10sInllYXIiOiIyMDIyIiwicmVjb3JkU3RhdHVzIjoiYWN0aXZlIiwiZ3VpZCI6IjA4ZWM3NjBhLTFlZDItNDc0YS1hYTk3LTk2YzQ5ZTM0ZDYxZiIsInRpdGxlIjoiUHJlcGFyaW5nIGZNUkkgZGF0YSBmb3Igc3RhdGlzdGljYWwgYW5hbHlzaXMiLCJhdXRob3JzIjpbIk5pZXRvLUNhc3Rhbm9uLCBBbGZvbnNvIl0sInNlY29uZGFyeVRpdGxlIjoiYXJYaXYgcHJlcHJpbnQgYXJYaXY6MjIxMC4xMzU2NCIsInJlZmVyZW5jZVR5cGUiOiIxNyJ9XSwiZ3JvdXBHdWlkcyI6W10sImd1aWQiOiIwOGVjNzYwYS0xZWQyLTQ3NGEtYWE5Ny05NmM0OWUzNGQ2MWYifV19).

**First-level analysis.** ROI-to-ROI connectivity (RRC) matrices were estimated characterizing the functional connectivity between each pair of regions among 116 ROIs [(9)](https://web.endnote.com/citations/eyJkaXNwbGF5VGV4dCI6Iig4KSIsImNpdGF0aW9ucyI6W3siYmlibGlvQ29udGVudCI6W3siaXNibiI6IjEwNTMtODExOSIsInJlZmVyZW5jZVR5cGUiOiIxNyIsInBhZ2VzIjoiMS01IiwicmVjb3JkU3RhdHVzIjoiYWN0aXZlIiwiYXV0aG9ycyI6WyJSb2xscywgRWRtdW5kIFQiLCJKb2xpb3QsIE1hcmMiLCJUem91cmlvLU1hem95ZXIsIE5hdGhhbGllIl0sInNlY29uZGFyeVRpdGxlIjoiTmV1cm9pbWFnZSIsImdyb3VwR3VpZHMiOltdLCJ5ZWFyIjoiMjAxNSIsInRpdGxlIjoiSW1wbGVtZW50YXRpb24gb2YgYSBuZXcgcGFyY2VsbGF0aW9uIG9mIHRoZSBvcmJpdG9mcm9udGFsIGNvcnRleCBpbiB0aGUgYXV0b21hdGVkIGFuYXRvbWljYWwgbGFiZWxpbmcgYXRsYXMiLCJ2b2x1bWUiOiIxMjIiLCJndWlkIjoiMzM5OTZlNjItMTRlYS00NTYwLWJlMjYtMmEzZjBkNGYxZTExIn1dLCJncm91cEd1aWRzIjpbXSwiZ3VpZCI6IjMzOTk2ZTYyLTE0ZWEtNDU2MC1iZTI2LTJhM2YwZDRmMWUxMSJ9XX0%3D). Functional connectivity strength was represented by Fisher-transformed bivariate correlation coefficients from a general linear model (weighted-GLM[(1)](https://web.endnote.com/citations/eyJkaXNwbGF5VGV4dCI6IigxKSIsImNpdGF0aW9ucyI6W3siYmlibGlvQ29udGVudCI6W3sieWVhciI6IjIwMjAiLCJpc2JuIjoiMDU3ODY0NDAwMiIsInJlZmVyZW5jZVR5cGUiOiI2IiwiYXV0aG9ycyI6WyJOaWV0by1DYXN0YW5vbiwgQWxmb25zbyJdLCJncm91cEd1aWRzIjpbXSwidGl0bGUiOiJIYW5kYm9vayBvZiBmdW5jdGlvbmFsIGNvbm5lY3Rpdml0eSBtYWduZXRpYyByZXNvbmFuY2UgaW1hZ2luZyBtZXRob2RzIGluIENPTk4iLCJndWlkIjoiNWZjNzA3MDAtZjE0Zi00MjkyLWExMGYtN2UyNDhmNjExODE4IiwicmVjb3JkU3RhdHVzIjoiYWN0aXZlIiwicHVibGlzaGVyIjoiSGlsYmVydCBQcmVzcyJ9XSwiZ3JvdXBHdWlkcyI6W10sImd1aWQiOiI1ZmM3MDcwMC1mMTRmLTQyOTItYTEwZi03ZTI0OGY2MTE4MTgifV19)), estimated separately for each pair of ROIs, characterizing the association between their BOLD signal time series. In order to compensate for possible transient magnetization effects at the beginning of each run, individual scans were weighted by a step function convolved with an SPM canonical hemodynamic response function and rectified.

**Supplementary Table 4.** Structural MRI acquisition protocols.

| **Scanner** | **Scanner Model** | **Sequence name** | **TR**  **(ms)** | **TE**  **(ms)** | **Flip angle**  **(°)** | **No. slices** | **Matrix dimensions** | **Voxel size**  **(mm)** |
| --- | --- | --- | --- | --- | --- | --- | --- | --- |
| 1 | GE Signa HDxt 1.5T | SAG T1 3D | 6500 | 2.78 | 12 | 106 | 512 x 623x 106 | 0.5 x 0.5 x 0.5 |
| 2 | Philips Achieva 3T | TFE | 7620 | 3.70 | 8 | 256 | 170 x 240 x 240 | 1 x 1 x 1 |
| 3 | Philips Ingenia 3T | T1 3D FFE | 7780 | 3.60 | 8 | 576 | 355 x 576 x 576 | 0.5 x 0.4 x 0.4 |
| 4 | Philips Ingenia Elition X 3T | T1 3D FFE | 7780 | 3.55 | 8 | 576 | 355 x 576 x 576 | 0.5 x 0.4 x 0.4 |
| 5 | Siemens Biograph_mMR 3T | MP RAGE | 2300 | 2.9 | 9 | 256 | 176 x 240 x 256 | 1 x 1 x 1 |
| 6 | Siemens Magnetom Lumina 3T | MP RAGE | 1800 | 2.13 | 8 | 256 | 192 x 256 x 256 | 1 x 1 x 1 |
| 7 | Siemens PrismaFit 3T | MP RAGE | 2300 | 2 | 9 | 256 | 160 x 240 x 256 | 1 x 1 x 1 |
| 8 | Siemens Skyra 3T | MP RAGE | 1700 | 2.25 | 8 | 224 | 208 x 224 x 224 | 1 x 1 x 1 |
| 9 | Siemens Spectra 3T | MP RAGE | 1900 | 2.42 | 9 | 256 | 176 x 256 x 256 | 1 x 1 x 1 |
| 10 | Siemens TrioTim 3T | MP RAGE | 2300 | 2 | 9 | 256 | 160 x 240 x 256 | 1 x 1 x 1 |
| 11 | Siemens Verio 3T | MP RAGE | 2300 | 2.9 | 9 | 256 | 176 x 240 x 256 | 1 x 1 x 1 |

| **Scanner** | **Scanner Model** | **TR (ms)** | **TE (ms)** | **Voxel size (mm)** | **No. volumes** | **Matrix dimension** | **Flip angle (°)** | **No. slices** | **Duration (min)** |
| --- | --- | --- | --- | --- | --- | --- | --- | --- | --- |
| 1 | GE Signa HDxt 1.5T | 2500 | 30 | 3.7 x 3.7 x 5 | 120 | 64 x 64 | 50 | 33 | 5 |
| 2 | Philips Achieva 3T | 3000 | 30 | 3 x 3 x 3 | 160 | 80 x 80 | 90 | 40 | 8 |
| 3 | Philips Ingenia 3T | 4970 | 30 | 2.5 x 2.5 x 2.7 | 121 | 96 x 96 | 82 | 45 | 10 |
| 4 | Philips Ingenia Elition X 3T | 4970 | 30 | 2.5 x 2.5 x 2.7 | 121 | 96 x 96 | 82 | 45 | 10 |
| 5 | Siemens Biograph_mMR 3T | 3000 | 30 | 3.4 x 3.4 x 3.4 | 197 | 64 x 64 | 90 | 48 | 10 |
| 6 | Siemens Magnetom Lumina 3T | 2500 | 30 | 2.5 x 2.5 x 2.5 | 235 | 94 x 94 | 94 | 66 | 10 |
| 7 | Siemens PrismaFit 3T | 2000 | 32 | 2.2 x 2.2 x 2.2 | 560 | 96 x 96 | 45 | 66 | 8 |
| 8 | Siemens Skyra 3T | 2660 | 30 | 3 x 3 x 3 | 300 | 76 x 76 | 90 | 46 | 10 |
| 9 | Siemens TrioTim 3T | 2000 | 27 | 2.5 x 2.5 x 3.5 | 240 | 92 x 92 | 80 | 36 | 8 |
| 10 | Siemens Verio 3T | 3000 | 30 | 3.4 x 3.4 x 3.4 | 197 | 64 x 64 | 90 | 48 | 10 |

**Supplementary Table 5.** Resting-state fMRI protocol information.

**Supplementary results**

**Structural neuroimaging results**

**Patterns of brain atrophy**

**Supplementary Table 6.** Atrophy patterns in FTLD combined compared to healthy controls.

|  |  |  |  |  | **MNI** |  |  |  |
| --- | --- | --- | --- | --- | --- | --- | --- | --- |
| **Peak region** | **Association regions** | **Side** | **Size** | **X** | **Y** | **Z** | **t** | **FWE *p*** |
| Hippocampus | Putamen, insula | L | 376724 | -32 | -14 | -12 | 21.30 | <.001 |
| Insula | Inferior frontal gyrus | L | - | -36 | 14 | 4 | 21.01 | <.001 |
| Insula | Orbitofrontal cortex | L | - | -34 | 21 | -9 | 20.96 | <.001 |

Note. Whole brain voxelwise, FWE, *p* <.001, 100 contiguous voxels.

**Behavioural-variant frontotemporal dementia (bvFTD)**

**Supplementary Table 7.** Atrophy patterns in bvFTD compared to healthy controls.

|  |  |  |  |  | **MNI** |  |  |  |
| --- | --- | --- | --- | --- | --- | --- | --- | --- |
| **Peak region** | **Association regions** | **Side** | **Size** | **X** | **Y** | **Z** | **t** | ****p*** |
| Rectus | Orbitofrontal cortex (medial), insula | L | 59428 | -2 | 38 | -16 | 5.24 | <0.001 |
| Orbitofrontal cortex | Insula, rectus, | R | - | 8 | 45 | -12 | 4.96 | <0.001 |
| Middle cingulate cortex | Paracentral lobule | L | - | -3 | -26 | 40 | 4.93 | <0.001 |
| Postcentral gyrus | Parietal cortex (inferior and superior) | R | 1212 | 39 | -38 | 56 | 4.40 | <0.001 |
| Parietal cortex (inferior) | Angular gyrus, parietal cortex (superior) | R | - | 38 | -51 | 51 | 3.85 | <0.001 |
| Occipital (mid) | Occipital (superior), cuneus | L | 477 | -28 | -86 | 24 | 4.04 | <0.001 |
| Occipital (mid) | Occipital (superior), cuneus | L | - | -24 | -90 | 15 | 3.54 | <0.001 |
| Postcentral gyrus | Precentral gyrus, supramarginal gyrus | L | 814 | -52 | -12 | 30 | 4.00 | <0.001 |
| Middle frontal gyrus | Inferior frontal gyrus, superior frontal gyrus | R | 620 | 42 | 45 | 14 | 3.91 | <0.001 |
| Middle frontal gyrus | Inferior frontal gyrus, superior frontal gyrus | R | - | 40 | 40 | 32 | 3.82 | <0.001 |
| Occipital (mid) | Middle temporal gyrus, occipital (superior) | R | 210 | 39 | -80 | 18 | 3.88 | <0.001 |
| Middle temporal gyrus | Superior temporal gyrus, inferior temporal gyrus | R | 1272 | 62 | -28 | -2 | 3.86 | <0.001 |
| Middle temporal gyrus | Superior temporal gyrus, inferior temporal gyrus | R | - | 62 | -24 | -15 | 3.76 | <0.001 |
| Middle temporal gyrus | Superior temporal gyrus, supramarginal gyrus | R | - | 62 | -48 | 12 | 3.68 | <0.001 |
| Precentral gyrus | Postcentral gyrus, superior frontal gyrus | R | 167 | 40 | -16 | 58 | 3.84 | <0.001 |
| Postcentral gyrus | Parietal (inferior), supramarginal gyrus | L | 286 | -50 | -33 | 50 | 3.69 | <0.001 |
| Precentral gyrus | Frontal inferior operculum | R | 236 | 51 | -3 | 33 | 3.66 | <0.001 |
| Middle temporal gyrus | Occipital (middle and inferior) | L | 291 | -45 | -69 | 9 | 3.57 | <0.001 |
| Middle temporal gyrus | Inferior temporal gyrus, occipital (inferior) | L | - | -51 | -64 | -3 | 3.53 | <0.001 |
| Postcentral gyrus | Precentral gyrus, parietal (inferior) | L | 171 | -34 | -22 | 52 | 3.57 | <0.001 |
| Superior temporal gyrus | Supramarginal gyrus, rolandic operculum | R | 261 | 58 | -33 | 18 | 3.55 | <0.001 |
| Occipital (mid) | Parietal (inferior), occipital (inferior) | L | 120 | -28 | -76 | 40 | 3.53 | <0.001 |
| Precentral gyrus | Paracentral lobule, Supplementary motor area | R | 149 | 16 | -26 | 69 | 3.48 | <0.001 |
| Occipital | - | R | 157 | 26 | -88 | 27 | 3.44 | <0.001 |
| Cuneus | - | R | - | 14 | -82 | 34 | 3.27 | 0.001 |
| Occipital | - | R | - | 30 | -87 | 16 | 3.11 | 0.001 |

Note. Whole brain voxelwise, **p* <.001 uncorrected. 100 contiguous voxels.

**Nonfluent variant primary progressive aphasia (nfvPPA)**

**Supplementary Table 8.** Atrophy patterns in nonfluent primary progressive aphasias compared to healthy controls.

|  |  |  |  |  | **MNI** |  |  |  |
| --- | --- | --- | --- | --- | --- | --- | --- | --- |
| **Peak region** | **Association regions** | **Side** | **Size** | **X** | **Y** | **Z** | **t** | ****p*** |
| Postcentral | Precentral gyrus, parietal (inferior) | L | 29361 | -51 | -8 | 38 | 5.68 | <0.001 |
| Superior temporal gyrus | Heschl’s gyrus, rolandic operculum, insula | L | - | -63 | -14 | 9 | 5.00 | <0.001 |
| Superior temporal gyrus | Heschl’s gyrus, rolandic operculum, insula | L | - | -50 | -10 | 3 | 4.84 | <0.001 |
| Supplementary motor | Superior frontal gyrus | L | 13193 | -4 | 12 | 51 | 5.57 | <0.001 |
| Precuneus | Calcarine | L | - | -2 | -63 | 22 | 4.65 | <0.001 |
| Middle cingulate cortex | Superior frontal gyrus (medial), anterior cingulate cortex | L | - | -4 | 24 | 36 | 4.56 | <0.001 |
| Precentral gyrus | Frontal operculum (inferior) | R | 6554 | 52 | -3 | 33 | 4.78 | <0.001 |
| Rolandic operculum | Heschl’s gyrus, insula, superior temporal gyrus | R | - | 58 | -6 | 9 | 4.30 | <0.001 |
| Postcentral gyrus | Precentral gyrus, rolandic operculum | R | - | 64 | 0 | 21 | 4.27 | <0.001 |
| Inferior temporal gyrus | Cerebellum | L | 1905 | -45 | -50 | -24 | 4.30 | <0.001 |
| Fusiform | Cerebellum, Occipital (inferior) | L | - | -28 | -72 | -14 | 3.77 | <0.001 |
| Fusiform | Cerebellum | L | - | -21 | -78 | -12 | 3.68 | <0.001 |
| Occipital (mid) | Middle temporal gyrus, Occipital (superior) | R | 697 | 42 | -81 | 16 | 4.14 | <0.001 |
| Occipital (mid) | Occipital (superior), cuneus | R | - | 28 | -86 | 12 | 3.28 | 0.001 |
| Occipital (mid) | Angular gyrus, Middle temporal gyrus | R | - | 46 | -72 | 27 | 3.27 | 0.001 |
| Middle frontal gyrus | Inferior frontal gyrus, superior frontal gyrus | L | 600 | -45 | 54 | 14 | 4.08 | <0.001 |
| Inferior frontal gyrus | Middle frontal gyrus | L | - | -42 | 45 | 2 | 3.99 | <0.001 |
| Fusiform | Lingual gyrus, cerebellum | R | 629 | 26 | -80 | -14 | 4.03 | <0.001 |
| Lingual gyrus | Cerebellum | R | - | 15 | -81 | -12 | 3.68 | <0.001 |
| Occipital (inferior) | Inferior temporal gyrus, middle temporal gyrus | L | 466 | -51 | -68 | -4 | 3.98 | <0.001 |
| Angular gyrus | Superior temporal gyrus, middle temporal gyrus | R | 499 | 57 | -57 | 24 | 3.94 | <0.001 |
| Middle temporal gyrus | Superior temporal gyrus, angular gyrus | R | - | 63 | -51 | 10 | 3.36 | <0.001 |
| Middle temporal gyrus | Superior temporal gyrus, angular gyrus | R | - | 58 | -64 | 12 | 3.34 | <0.001 |
| Occipital | - | R | 431 | 38 | -76 | 39 | 3.93 | <0.001 |
| Occipital | - | L | 471 | -27 | -86 | 24 | 3.88 | <0.001 |
| Occipital | - | L | - | -32 | -80 | 21 | 3.67 | <0.001 |
| Occipital | - | L | - | -24 | -90 | 15 | 3.51 | <0.001 |
| Thalamus | - | L | 122 | -2 | 4 | -2 | 3.66 | <0.001 |

Note. Whole brain voxelwise, **p* <.001 uncorrected. 100 contiguous voxels.

**Semantic variant Primary Progressive Aphasia (svPPA)**

**Supplementary Table 9.** Atrophy patterns in the semantic variant of primary progressive aphasia compared to healthy controls.

|  |  |  |  |  | **MNI** |  |  |  |
| --- | --- | --- | --- | --- | --- | --- | --- | --- |
| **Peak region** | **Association regions** | **Side** | **Size** | **X** | **Y** | **Z** | **t** | ****p*** |
| Fusiform | Inferior temporal gyrus, parahippocampal gyrus | L | 159034 | -33 | -9 | -39 | 10.08 | <0.001 |
| Temporal pole | Superior temporal gyrus, middle temporal gyrus | L | - | -45 | 9 | -21 | 9.71 | <0.001 |
| Temporal pole | Superior temporal gyrus, middle temporal gyrus | L | - | -46 | 2 | -16 | 9.65 | <0.001 |
| Occipital (mid) | Middle temporal gyrus, occipital (superior) | R | 1215 | 42 | -81 | 18 | 4.11 | <0.001 |
| Occipital (mid) | Middle temporal gyrus, angular gyrus | R | - | 51 | -80 | 24 | 3.72 | <0.001 |
| Occipital (mid) | Occipital (superior), cuneus | R | - | 26 | -84 | 10 | 3.71 | <0.001 |
| Parietal (superior) | Parietal (inferior), postcentral gyrus | L | 936 | -30 | -52 | 57 | 3.97 | <0.001 |
| Parietal (inferior) | Angular gyrus, occipital (middle) | L | - | -33 | -51 | 40 | 3.81 | <0.001 |
| Parietal (inferior) | Postcentral gyrus, angular gyrus | L | - | -32 | -42 | 42 | 3.73 | <0.001 |
| Postcentral gyrus | Parietal (inferior and superior) | R | 892 | 40 | -36 | 56 | 3.89 | <0.001 |
| Parietal (inferior) | Supramarginal gyrus, postcentral gyrus | R | - | 51 | -36 | 50 | 3.86 | <0.001 |
| Parietal (inferior) | Supramarginal gyrus, parietal (superior) | R | - | 56 | -45 | 51 | 3.53 | <0.001 |
| Parietal (superior) | Angular gyrus, parietal (inferior) | R | 371 | 38 | -57 | 54 | 3.67 | <0.001 |
| Angular gyrus | Parietal (superior and inferior) | R | - | 32 | -62 | 48 | 3.59 | <0.001 |
| Parietal (inferior) | Angular gyrus, parietal (superior) | R | - | 44 | -57 | 48 | 3.57 | <0.001 |
| Postcentral gyrus | Parietal (inferior), supramarginal gyrus | L | 158 | -51 | -33 | 51 | 3.53 | <0.001 |
| Postcentral gyrus | Precentral, supramarginal gyrus | L | 256 | -52 | -12 | 30 | 3.49 | <0.001 |
| Postcentral gyrus | Parietal (inferior), supramarginal gyrus | L | - | -50 | -15 | 38 | 3.31 | 0.001 |

Note. Whole brain voxelwise, **p* <.001 uncorrected. 100 contiguous voxels.

**Corticobasal syndrome, progressive supranuclear palsy, FTD with motor neuron disease (CBS, PSP, FTD-MND)**

**Supplementary Table 10.** Atrophy patterns in FTD-mixed, including corticobasal syndrome, progressive supranuclear palsy and FTD-with motor neuron disease features compared to healthy controls.

|  |  |  |  |  | **MNI** |  |  |  |
| --- | --- | --- | --- | --- | --- | --- | --- | --- |
| **Peak region** | **Association regions** | **Side** | **Size** | **X** | **Y** | **Z** | **t** | ****p*** |
| Cerebellum | Inferior temporal gyrus, fusiform gyrus | L | 1999 | -46 | -51 | -30 | 4.54 | <0.001 |
| Cerebellum | - | L | - | -54 | -48 | -42 | 4.13 | <0.001 |
| Angular gyrus | Parietal (inferior), occipital (middle) | L | 901 | -33 | -52 | 36 | 4.24 | <0.001 |
| Parietal (superior) | Parietal (inferior), postcentral gyrus | L | - | -30 | -50 | 57 | 3.33 | <0.001 |
| Parietal (inferior) | Postcentral gyrus, parietal (superior) | L | - | -30 | -44 | 46 | 3.29 | 0.001 |
| Precuneus | Parietal (superior), paracentral lobule | R | 2657 | 10 | -52 | 57 | 4.23 | <0.001 |
| Paracentral lobule | Precuneus, middle cingulate cortex | L | - | -9 | -38 | 63 | 4.17 | <0.001 |
| Precuneus | Parietal (superior), postcentral gyrus | L | - | -9 | -52 | 66 | 3.89 | <0.001 |
| Postcentral gyrus | Rolandic operculum, precentral gyrus, insula | R | 1180 | 68 | 2 | 15 | 4.15 | <0.001 |
| Rolandic operculum | Superior temporal gyrus, Heschl’s gyrus | R | - | 66 | -6 | 9 | 4.07 | <0.001 |
| Precentral gyrus | Frontal operculum (inferior), rolandic operculum | R | - | 64 | 12 | 14 | 3.54 | <0.001 |
| Supplementary Motor | Superior frontal gyrus | R | 945 | 6 | -3 | 66 | 3.96 | <0.001 |
| Supplementary Motor | Superior frontal gyrus | L | - | -4 | -2 | 66 | 3.79 | <0.001 |
| Supplementary Motor | Superior frontal gyrus | L | - | -8 | 8 | 72 | 3.42 | <0.001 |
| Middle frontal gyrus | Inferior frontal gyrus, superior frontal gyrus | L | 141 | -44 | 56 | 15 | 3.76 | <0.001 |
| Postcentral gyrus | Precentral gyrus, rolandic operculum, insula | R | 168 | 44 | -34 | 54 | 3.75 | <0.001 |
| Superior frontal gyrus | Middle frontal gyrus | L | 336 | -24 | 39 | 27 | 3.68 | <0.001 |
| Postcentral gyrus | Precentral gyrus, Rolandic operculum | L | 555 | -51 | -14 | 28 | 3.67 | <0.001 |
| Precentral gyrus | Postcentral gyrus, parietal (inferior) | L | - | -42 | -10 | 40 | 3.67 | <0.001 |
| Postcentral gyrus | Postcentral gyrus, parietal (inferior) | L | - | -36 | -24 | 39 | 3.27 | 0.001 |
| Supplementary motor | Paracentral lobule | R | 141 | 2 | -22 | 54 | 3.52 | <0.001 |
| Cerebellum | - | R | 130 | 45 | -56 | -40 | 3.47 | <0.001 |
| Cerebellum | - | R | - | 54 | -60 | -40 | 3.19 | 0.001 |

Note. Whole brain voxelwise, **p* <.001 uncorrected. 100 contiguous voxels.

**
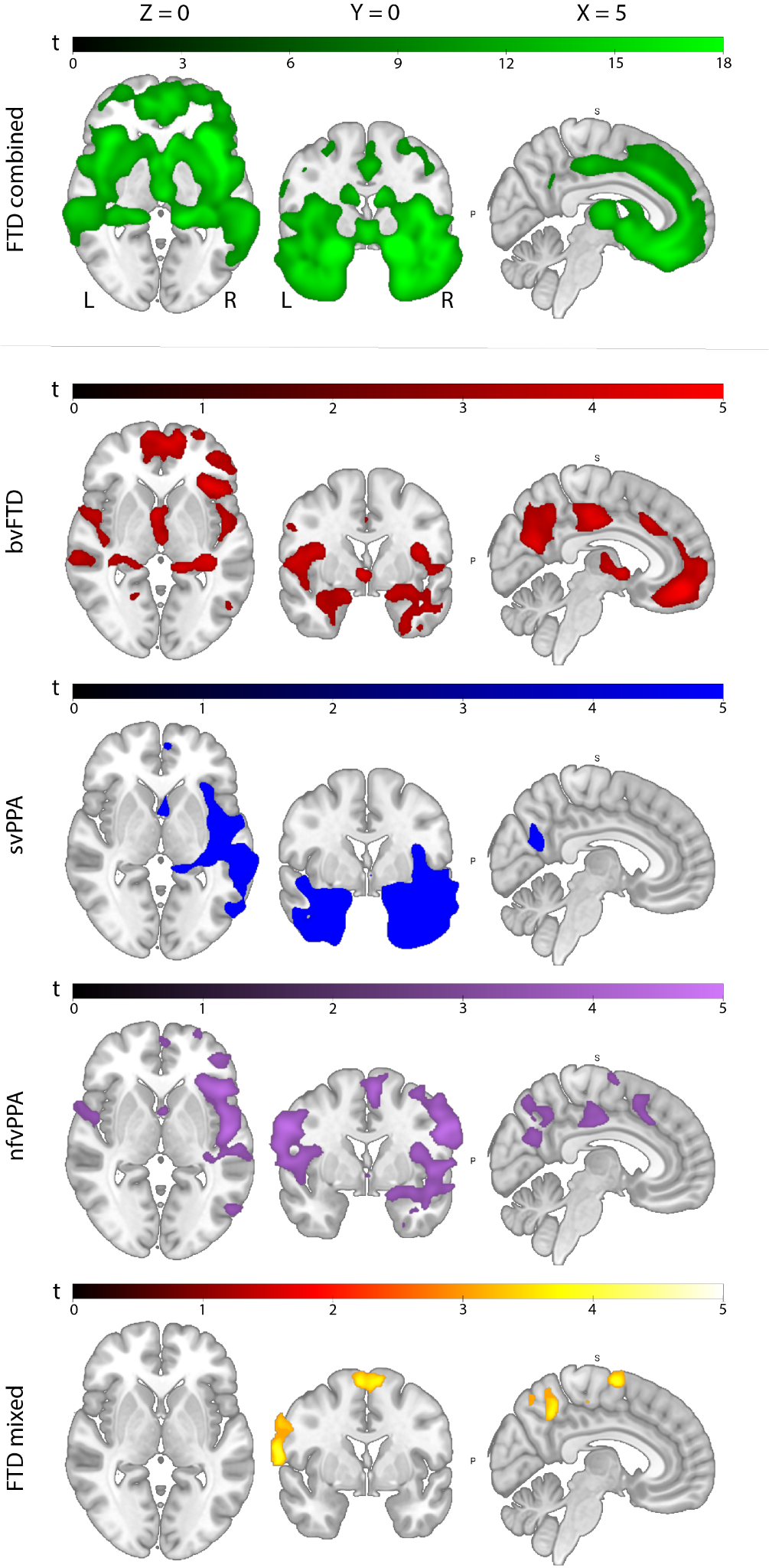
**

**Supplementary Figure 1.** Atrophy compared to controls in FTLD vs controls (p-FWE, *p*<.001), and in subtypes (*p*-uncorr<0.001).

**Alzheimer’s disease subtypes**


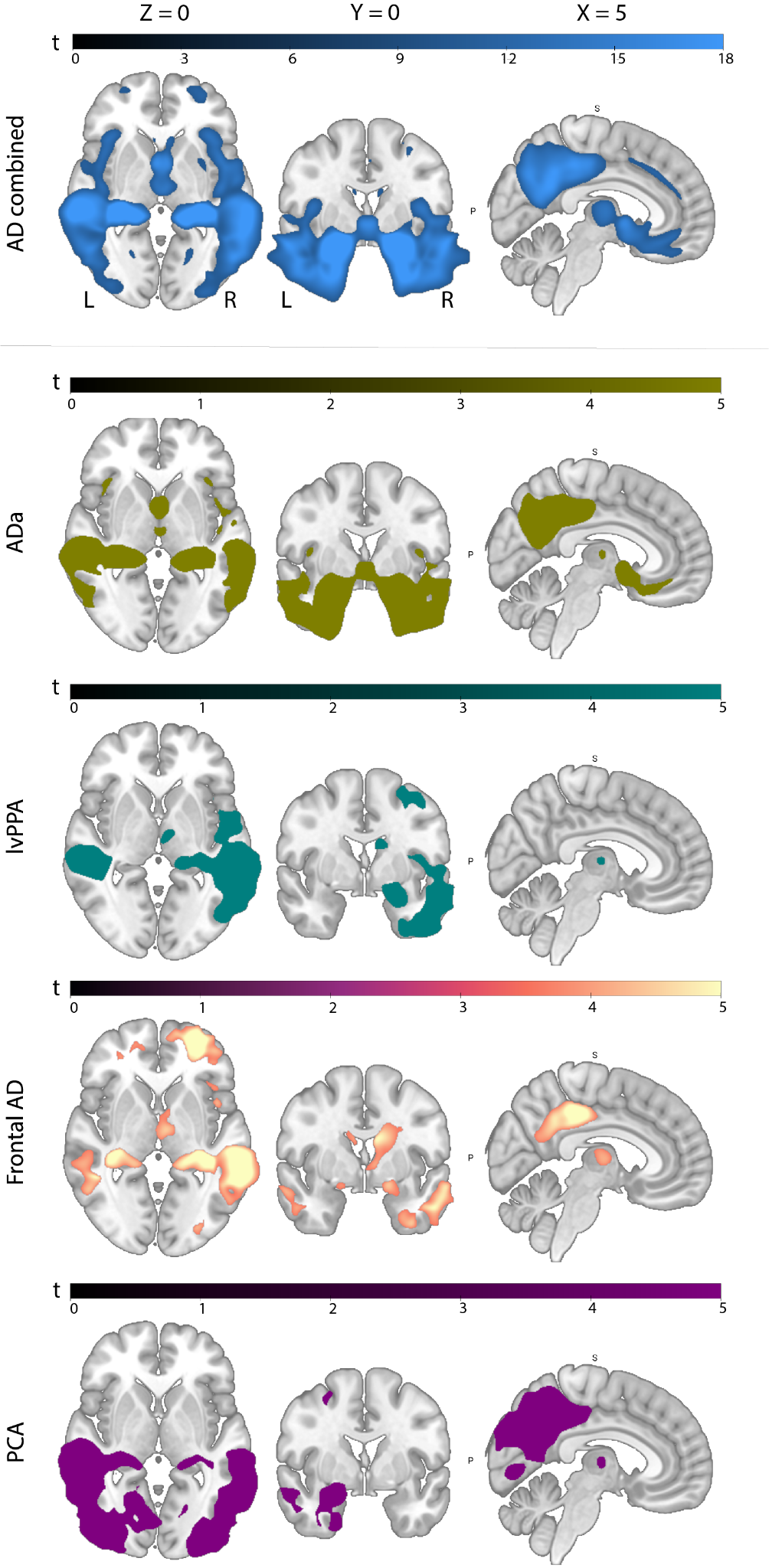


**Supplementary Figure 2.** Atrophy compared to controls in AD vs controls (p-FWE, *p*<.001), and in subtypes (*p*-uncorr<0.001)

**Supplementary Table 11.** Atrophy patterns in Alzheimer’s disease combined compared to healthy controls.

|  |  |  |  |  | **MNI** |  |  |  |
| --- | --- | --- | --- | --- | --- | --- | --- | --- |
| **Peak region** | **Association regions** | **Side** | **Size** | **X** | **Y** | **Z** | **t** | **FDR *p*** |
| Amygdala | Parahippocampal gyrus, olfactory cortex, temporal pole, superior temporal gyrus, putamen, orbitofrontal cortex (medial and posterior), rectus, hippocampus, insula, thalamus, anterior and posterior cingulate cortex, and precuneous bilaterally | R | 43986 | 21 | 4 | -18 | 7.65 | 0.001 |
| Rectus | Orbitofrontal cortex, superior frontal gyrus | L | - | -9 | 30 | -24 | 7.52 | 0.001 |
| Amygdala | Olfactory cortex, temporal pole, superior temporal gyrus, Insula, orbitofrontal cortex (medial and posterior), parahippocampal gyrus, putamen, rectus | L | - | -24 | 4 | -18 | 7.43 | 0.001 |
| Middle frontal gyrus | Superior frontal gyrus, inferior frontal gyrus, frontal pole | L | 168 | -44 | 56 | -2 | 2.35 | 0.015 |
| Middle frontal gyrus | Orbitofrontal cortex (lateral and anterior), inferior frontal gyrus, superior frontal gyrus, frontal pole | L | - | -42 | 50 | -9 | 2.34 | 0.017 |

Note. All Alzheimer’s presentations included (typical and atypical). All clusters are reported at TFCE, FDR-corrected *p* < .05, 50 contiguous voxels threshold. Peak region relates to the coordinates displayed in the table; associate regions are within the cluster. Covariates included group (AD vs control), scanner, and TIV.

**Alzheimer’s Disease subtypes**

**Alzheimer’s Disease (amnestic)**

**Supplementary Table 12.** Atrophy patterns in Alzheimer’s disease with amnestic features (typical AD) compared to healthy controls.

|  |  |  |  |  | **MNI** |  |  |  |
| --- | --- | --- | --- | --- | --- | --- | --- | --- |
| **Peak region** | **Association regions** | **Side** | **Size** | **X** | **Y** | **Z** | **t** | ****p*** |
| **Amygdala** | **Putamen** | R | 678510 | 27 | -9 | -14 | 25.76 | <.001 |
| Hippocampus | Amygdala, putamen | L |  | -27 | -10 | -14 | 25.73 | <.001 |
| Hippocampus | Parahippocampal gyrus, thalamus | L |  | -27 | -32 | -8 | 23.15 | <.001 |
| Cerebellum | - | R | 1330 | 8 | -60 | -70 | 4.82 | <.001 |

Note. Whole brain voxelwise, **p* <.001 uncorrected. 100 contiguous voxels.

**Atypical Alzheimer’s Disease**

**Logopenic-variant of Primary Progressive Aphasia**

**Supplementary Table 13.** Atrophy patterns in logopenic-variant primary progressive aphasia compared to healthy controls.

|  |  |  |  |  | **MNI** |  |  |  |
| --- | --- | --- | --- | --- | --- | --- | --- | --- |
| **Peak region** | **Association regions** | **Side** | **Size** | **X** | **Y** | **Z** | **t** | ****p*** |
| Middle temporal gyrus | Angular gyrus, superior temporal gyrus | L | 292468 | -58 | -58 | 15 | 12.02 | <0.001 |
| Inferior temporal gyrus | Temporal fusiform, occipital (inferior) | L | - | -51 | -52 | -18 | 11.51 | <0.001 |
| Middle temporal gyrus | Superior temporal gyrus, Inferior temporal gyrus | L | - | -64 | -26 | -4 | 11.39 | <0.001 |
| Anterior cingulate cortex | Orbitofrontal cortex (medial), rectus | R | 490 | 16 | 33 | -8 | 3.80 | <0.001 |
| Orbitofrontal cortex | Middle frontal gyrus, orbitofrontal cortex | R | - | 28 | 39 | -14 | 3.51 | <0.001 |
| Orbitofrontal cortex | Superior frontal gyrus, Orbitofrontal cortex | R | - | 20 | 48 | -9 | 3.33 | <0.001 |

Note. Whole brain voxelwise, **p* <.001 uncorrected. 100 contiguous voxels.

**Frontal-variant Alzheimer’s Disease**

**Supplementary Table 14.** Atrophy patterns of frontal-variant Alzheimer’s Disease compared to healthy controls.

|  |  |  |  |  | **MNI** |  |  |  |
| --- | --- | --- | --- | --- | --- | --- | --- | --- |
| **Peak region** | **Association regions** | **Side** | **Size** | **X** | **Y** | **Z** | **t** | ****p*** |
| Middle frontal gyrus | Superior frontal gyrus, inferior frontal gyrus | L | 107452 | -32 | 57 | 12 | 7.18 | <.001 |
| Middle temporal gyrus | Inferior temporal gyrus, superior temporal gyrus | L | - | -44 | -21 | -14 | 7.05 | <.001 |
| Middle temporal gyrus | Inferior temporal gyrus, superior temporal gyrus | L | - | -57 | -30 | -9 | 6.87 | <.001 |
| Insula | Inferior frontal gyrus, inferior frontal operculum | R | 177 | 39 | 21 | 3 | 3.51 | <.001 |
| Insula | Inferior frontal operculum, putamen | R | - | 38 | 8 | 8 | 3.39 | <.001 |

Note. Whole brain voxelwise, **p* <.001 uncorrected. 100 contiguous voxels.

**Posterior cortical atrophy**

**Supplementary Table 15.** Atrophy patterns of posterior cortical atrophy compared to healthy controls.

|  |  |  |  |  | **MNI** |  |  |  |
| --- | --- | --- | --- | --- | --- | --- | --- | --- |
| **Peak region** | **Association regions** | **Side** | **Size** | **X** | **Y** | **Z** | **t** | ****p*** |
| Temporal fusiform | Cerebellum, occipital (inferior) | R | 382446 | 28 | -76 | -15 | 12.18 | <0.001 |
| Occipital (superior) | Occipital (mid), cuneus | R | - | 30 | -86 | 26 | 10.71 | <0.001 |
| Temporal fusiform | Occipital (inferior), lingual | R | - | 33 | -60 | -9 | 10.68 | <0.001 |

Note. Whole brain voxelwise, **p* <.001 uncorrected. 100 contiguous voxels.

**Structural correlates associated with cardiovascular risk**

**Supplementary Table 16.** Structural correlates of cardiovascular risk in FTLD.

|  |  |  |  |  | **MNI** |  |  |  |
| --- | --- | --- | --- | --- | --- | --- | --- | --- |
| **Peak region** | **Association regions** | **Side** | **Size** | **X** | **Y** | **Z** | **t** | **FDR *p*** |
| Insula | Heschl’s gyrus, orbitofrontal cortex, middle frontal gyrus, amygdala, hippocampus, parahippocampus, thalamus, posterior cingulate cortex, planum polare, temporal pole | R | 8693 | 45 | -15 | 9 | 7.14 | 0.002 |
| Insula | Superior temporal gyrus, rolandic operculum, planum polare | R | - | 51 | 2 | -2 | 5.65 | 0.002 |
| Hippocampus | Thalamus, parahippocampus | R | - | 36 | -36 | -4 | 5.54 | 0.002 |
| Hippocampus | Parahippocampal gyrus, thalamus, amygdala, insula, | L | 2634 | -21 | -42 | -8 | 5.99 | 0.002 |
| Hippocampus | Parahippocampal gyrus, thalamus, amygdala | L | - | -34 | -39 | -4 | 4.96 | 0.002 |
| Hippocampus | Thalamus, parahippocampal gyrus | L | - | -15 | -38 | 0 | 4.91 | 0.002 |
| Mid Cingulate cortex | Anterior cingulate cortex, posterior cingulate cortex, orbitofrontal cortex bilaterally | R | 9280 | 2 | -24 | 46 | 5.76 | 0.002 |
| Mid Cingulate cortex | Posterior cingulate cortex, anterior cingulate cortex bilaterally | R | - | 6 | -16 | 48 | 5.54 | 0.002 |
| Mid Cingulate cortex | Anterior cingulate cortex bilaterally | R | - | 2 | 20 | 38 | 5.04 | 0.002 |
| Insula | Rolandic operculum | L | 325 | -38 | -22 | 14 | 4.81 | 0.002 |
| Insula | Parietal operculum | L | - | -36 | -28 | 20 | 4.23 | 0.002 |
| Insula | Heschl’s gyrus, planum polare | L | - | -46 | -10 | 4 | 3.89 | 0.002 |
| Orbitofrontal cortex | Inferior frontal gyrus, insula | L | 210 | -52 | 20 | -4 | 4.08 | 0.002 |
| Orbitofrontal cortex | Temporal pole | L | - | -42 | 18 | -15 | 2.39 | 0.028 |
| Superior frontal gyrus | - | R | 172 | 28 | 64 | -2 | 3.14 | 0.007 |
| Insula | Frontal operculum cortex | L | 106 | -40 | 20 | 4 | 2.67 | 0.015 |
| Superior frontal gyrus | - | R | 66 | 6 | 62 | -22 | 2.49 | 0.028 |
| Superior frontal gyrus | - | R | - | 15 | 66 | -18 | 2.28 | 0.038 |
| Middle frontal gyrus | Orbitofrontal cortex | L | 114 | -45 | 50 | -10 | 2.43 | 0.028 |
| Middle frontal gyrus | - | L | - | -45 | 56 | -4 | 2.41 | 0.022 |
| Inferior frontal gyrus | - | R | 70 | 36 | 39 | -20 | 2.26 | 0.031 |

Note. All FTLD subtypes included. All clusters are reported at TFCE, FDR-corrected *p* < .05, 50 contiguous voxels threshold. Peak region relates to the coordinates displayed in the table; associate regions are within the cluster. Covariates included group (FTLD vs control), scanner, and TIV.

**FTLD subtypes**

We repeated our main analyses investigating cardiovascular risk while controlling for FTD subtypes (i.e., bvFTD, nfvPPA, svPPA, and CBS/PSP or FTD MND) to ensure that our results were not driven by the combination of FTD subtypes. Here, we replicated our main results, showing that increased cardiovascular risk was associated with AIN structures such as the bilateral insula, thalamus, amygdala, and hippocampus (Supplementary Table 17).

**Supplementary Table 17.** Brain regions associated with cardiovascular risk in FTLD, controlling for FTLD subtype, scanner, and TIV.

|  |  |  |  |  | **MNI** |  |  |  |
| --- | --- | --- | --- | --- | --- | --- | --- | --- |
| **Peak region** | **Association regions** | **Side** | **Size** | **X** | **Y** | **Z** | **t** | **FDR *p*** |
| Insula | Heschl’s gyrus, rolandic operculum | R | 9075 | 45 | -15 | 9 | 6.95 | 0.002 |
| Hippocampus | Thalamus, amygdala, parahippocampal gyrus, | R | - | 16 | -33 | -4 | 5.47 | 0.002 |
| Hippocampus | Thalamus | R | - | 36 | -36 | -4 | 5.39 | 0.002 |
| Hippocampus | Parahippocampal gyrus, thalamus | L | 2651 | -21 | -42 | -8 | 5.93 | 0.002 |
| Hippocampus | Parahippocampus | L | - | -34 | -39 | -4 | 4.78 | 0.002 |
| Hippocampus | Thalamus | L | - | -15 | -38 | 0 | 4.75 | 0.002 |
| Mid cingulate cortex | Anterior cingulate cortex, posterior cingulate cortex | R | 9433 | 2 | -24 | 46 | 5.81 | 0.002 |
| Mid cingulate cortex | Anterior cingulate cortex, posterior cingulate cortex | R | - | 6 | -16 | 48 | 5.49 | 0.002 |
| Mid cingulate cortex | Anterior cingulate cortex, posterior cingulate cortex | R | - | 2 | 20 | 38 | 4.93 | 0.002 |
| Insula | Rolandic operculum, parietal operculum | L | 277 | -38 | -22 | 14 | 4.49 | 0.002 |
| Insula | Parietal operculum | L | - | -36 | -28 | 20 | 4.01 | 0.003 |
| Insula | Heschl’s gyrus | L | - | -46 | -10 | 4 | 3.59 | 0.004 |
| Inferior frontal gyrus | Orbitofrontal cortex, frontal operculum | L | 222 | -52 | 20 | -4 | 4.02 | 0.002 |
| Orbitofrontal cortex | Insula, superior temporal gyrus | L | - | -42 | 18 | -15 | 2.45 | 0.025 |
| Orbitofrontal cortex | - | R | 200 | 28 | 64 | -2 | 3.21 | 0.006 |
| Insula | Frontal operculum cortex, inferior frontal gyrus | L | 152 | -40 | 20 | 4 | 2.8 | 0.011 |
| Orbitofrontal cortex | - | L | 188 | -45 | 56 | -4 | 2.56 | 0.018 |
| Orbitofrontal cortex | - | L | - | -46 | 50 | -10 | 2.54 | 0.022 |
| Orbitofrontal cortex | - | L | - | -48 | 48 | -2 | 2.19 | 0.042 |
| Orbitofrontal cortex | - | R | 74 | 6 | 62 | -22 | 2.54 | 0.028 |
| Orbitofrontal cortex | - | R | - | 15 | 66 | -18 | 2.38 | 0.03 |

Note. All clusters are reported at TFCE, FDR-corrected *p* < .05, 50 contiguous voxels threshold. Regions labelled with AAL2 atlas. Covariates included group (FTD subtype vs control), scanner, and TIV.

**Supplementary Table 18.** Structural correlates associated with cardiovascular risk in AD.

|  |  |  |  |  | **MNI** |  |  |  |
| --- | --- | --- | --- | --- | --- | --- | --- | --- |
| **Peak region** | **Association regions** | **Side** | **Size** | **X** | **Y** | **Z** | **t** | **FDR *p*** |
| Amygdala | Parahippocampal gyrus, olfactory cortex, temporal pole, superior temporal gyrus, putamen, orbitofrontal cortex (medial and posterior), rectus, hippocampus, insula, thalamus, anterior and posterior cingulate cortex, and precuneous bilaterally | R | 43986 | 21 | 4 | -18 | 7.65 | 0.001 |
| Rectus | Orbitofrontal cortex, superior frontal gyrus | L | - | -9 | 30 | -24 | 7.52 | 0.001 |
| Amygdala | Olfactory cortex, temporal pole, superior temporal gyrus, Insula, orbitofrontal cortex (medial and posterior), parahippocampal gyrus, putamen, rectus | L | - | -24 | 4 | -18 | 7.43 | 0.001 |
| Middle frontal gyrus | Superior frontal gyrus, inferior frontal gyrus, frontal pole | L | 168 | -44 | 56 | -2 | 2.35 | 0.015 |
| Middle frontal gyrus | Orbitofrontal cortex (lateral and anterior), inferior frontal gyrus, superior frontal gyrus, frontal pole | L | - | -42 | 50 | -9 | 2.34 | 0.017 |

Note. All Alzheimer’s presentations included (typical and atypical). All clusters are reported at TFCE, FDR-corrected *p* < .05, 50 contiguous voxels threshold. Peak region relates to the coordinates displayed in the table; associate regions are within the cluster. Covariates included group (AD vs control), scanner, and TIV.

**AD subtypes**

We repeated our main analyses controlling for AD subtype (typical amnestic AD, and atypical AD variants: frontal-variant AD, posterior cortical atrophy, logopenic variant primary progressive aphasia). Here, we replicated our main results, with reduced grey matter integrity in the bilateral amygdala, hippocampus, parahippocampal gyrus, superior temporal gyrus, temporal pole, insula, thalamus, anterior cingulate and paracingulate cortex were associated with higher cardiovascular risk scores in AD, controlling for AD subtypes (Supplementary Table 19).

**Supplementary Table 19.** Brain regions associated with cardiovascular risk in AD, controlling for AD subtype, scanner, and TIV.

|  |  |  |  |  | **MNI** |  |  |  |
| --- | --- | --- | --- | --- | --- | --- | --- | --- |
| **Peak region** | **Association regions** | **Side** | **Size** | **X** | **Y** | **Z** | **t** | **FDR *p*** |
| Amygdala | Hippocampus, thalamus, parahippocampal gyrus, insula, Heschl’s gyrus, middle frontal gyrus, anterior cingulate cortex, posterior cingulate cortex | R | 45381 | 21 | 4 | -18 | 7.68 | 0.001 |
| Orbitofrontal cortex (superior) | Parahippocampal gyrus, Insula, thalamus, amygdala, hippocampus | L |  | -9 | 30 | -24 | 7.48 | 0.001 |
| Amygdala | Hippocampus, parahippocampal gyrus | L |  | -24 | 4 | -18 | 7.37 | 0.001 |
| Thalamus | - | R | 88 | 21 | -15 | -2 | 3.44 | 0.001 |
| Orbitofrontal cortex (middle) | - | L | 257 | -42 | 50 | -9 | 2.52 | 0.012 |
| Middle frontal gyrus | - | L |  | -44 | 56 | -2 | 2.5 | 0.010 |

Note. All clusters are reported at TFCE, FDR-corrected *p* < .05, 50 contiguous voxels threshold. Regions labelled with AAL2 atlas. Covariates included group (AD subtype vs control), scanner, and TIV.

Supplementary Table 20. Reduced structural integrity associated with greater cardiovascular risk in FTLD than in AD.

|  |  |  |  | **MNI** | | |  |  |
| --- | --- | --- | --- | --- | --- | --- | --- | --- |
| **Peak region** | **Associated regions** | **Side** | **Size** | **X** | **Y** | **Z** | ***t*** | ***FDR p*** |
| Insula | Heschl's gyrus | R | 164 | 42 | -12 | 6 | 5.15 | <.001 |
| Insula | OFC | R | - | 48 | 15 | -8 | 4.87 | <.001 |
| Insula | Rolandic operculum | R | - | 51 | 9 | -3 | 4.73 | <.001 |
| ACC | ACC | Bi | 129 | 0 | 51 | 3 | 4.98 | <.001 |
| ACC | ACC | L | - | -2 | 48 | 15 | 4.74 | <.001 |
| ACC | ACC | R | - | 2 | 40 | 24 | 4.16 | <.001 |

Note. All clusters are reported at TFCE, FDR-corrected p <.05, 50 contiguous voxels threshold. Peak region relates to the coordinates displayed in the table; associate regions are within the cluster. Covariates included TIV. Abbreviations: ACC, Anterior cingulate cortex; OFC, Orbitofrontal cortex.

**Functional neuroimaging results**

**Supplementary Table 21.** Reduced functional connectivity associated with increased cardiovascular risk in FTLD.

| **Cluster** | ***F*** | ***p*** | **Connections** | ***t*** | ***p*** |
| --- | --- | --- | --- | --- | --- |
| 1 | 17.06 | 0.001 | Insula R – Insula L | -4.13 | <0.001 |
| 2 | 15.16 | 0.002 | Thalamus R – Thalamus L | -3.89 | <0.001 |
| 3 | 4.45 | 0.022 | Parahippocampal L – Orbitofrontal (medial) R | -4.26 | <0.001 |
| - | - | - | Orbitofrontal (medial) R - Parahippocampal L | -4.26 | <0.001 |
| - | - | - | Orbitofrontal (medial) R - Parahippocampal R | -3.57 | 0.0012 |
| - | - | - | Parahippocampal R - Orbitofrontal (medial) R | -3.57 | 0.0012 |
| - | - | - | Parahippocampal L - Orbitofrontal (medial) L | -3.26 | 0.0060 |
| - | - | - | Orbitofrontal (medial) R – Hippocampus R | -3.02 | 0.0013 |
| - | - | - | Orbitofrontal (medial) L – Parahippocampal L | -3.26 | 0.0060 |
| - | - | - | Orbitofrontal (medial) R – Hippocampus L | -2.80 | 0.0027 |
| - | - | - | Hippocampus L – Orbitofrontal (medial) R | -2.80 | 0.0027 |
| - | - | - | Orbitofrontal (medial) L – Hippocampus R | -2.66 | 0.0040 |
| - | - | - | Hippocampus R – Orbitofrontal (medial) R | -3.02 | 0.0013 |
| - | - | - | Hippocampus R – Orbitofrontal (medial) L | -2.66 | 0.0041 |
| - | - | - | Parahippocampal R – Orbitofrontal (medial) L | -2.17 | 0.0152 |
| 4 | 3.96 | 0.038 | Parahippocampal L – Orbitofrontal (superior) R | -3.75 | <0.001 |
| - | - | - | Orbitofrontal (superior) R – Parahippocampal L | -3.75 | <0.001 |
| - | - | - | Parahippocampal L – Orbitofrontal (middle) R | -2.52 | 0.0060 |
| - | - | - | Parahippocampal L – Orbitofrontal (inferior) R | -2.36 | 0.0093 |
| - | - | - | Parahippocampal R – Orbitofrontal (superior) R | -2.55 | 0.0055 |
| 5 | 4.45 | 0.038 | Middle cingulate L – Posterior cingulate L | -2.83 | 0.0024 |
| - | - | - | Middle cingulate R – Posterior cingulate R | -2.66 | 0.0040 |
| 6 | 7.43 | 0.049 | Orbitofrontal (medial) R – Orbitofrontal (medial) L | -2.73 | 0.0030 |
| - | - | - | Orbitofrontal (medial) L – Orbitofrontal (medial) R | -2.73 | 0.0030 |

Note. ROI-to-ROI functional connectivity results are displayed. FDR p values shown at the cluster and ROI level.

**Supplementary Table 22.** Reduced functional connectivity associated with increased cardiovascular risk in AD.

| **Cluster** | ***F*** | ***p*** | **Connections** | ***t*** | ***p*** |
| --- | --- | --- | --- | --- | --- |
| 1 | 5.09 | 0.021 | Parahippocampal R - Orbitofrontal (medial) R | -3.94 | <0.001 |
|  | - | - | Orbitofrontal (medial) R - Parahippocampal R | -3.94 | <0.001 |
| - | - | - | Orbitofrontal (medial) R - Parahippocampal L | -3.67 | <0.001 |
| - | - | - | Parahippocampal L - Orbitofrontal (medial) R | -3.67 | <0.001 |
| - | - | - | Parahippocampal R - Orbitofrontal (medial) L | -3.39 | <0.001 |
| - | - | - | Parahippocampal L - Orbitofrontal (medial) L | -2.93 | 0.0017 |
| - | - | - | Orbitofrontal (medial) L - Parahippocampal R | -3.39 | <0.001 |
| - | - | - | Orbitofrontal (medial) R - Hippocampus R | -2.89 | 0.0020 |
| - | - | - | Orbitofrontal (medial) L - Parahippocampal L | -2.93 | 0.0017 |
| - | - | - | Hippocampus R - Orbitofrontal (medial) R | -2.89 | 0.0020 |
| 2 | 4.30 | 0.043 | Parahippocampal L - Orbitofrontal (middle) R | -3.65 | <0.001 |
| - | - | - | Parahippocampal R - Orbitofrontal (middle) R | -3.52 | <0.001 |
| - | - | - | Orbitofrontal (middle) R - Parahippocampal L | -3.65 | <0.001 |
| - | - | - | Orbitofrontal (middle) R - Parahippocampal R | -3.52 | <0.001 |
| - | - | - | Parahippocampal L - Orbitofrontal (superior) R | -3.09 | 0.0010 |
| - | - | - | Orbitofrontal (superior) R - Parahippocampal L | -3.09 | 0.0010 |

Note. ROI-to-ROI functional connectivity results are displayed. FDR p values shown at the cluster and ROI level.

[**Supplementary Reference list**](https://web.endnote.com/reference-list/)

1. D’Agostino Sr RB, Vasan RS, Pencina MJ, Wolf PA, Cobain M, Massaro JM, et al. (2008): General cardiovascular risk profile for use in primary care: the Framingham Heart Study. *Circulation*. 117:743-753.

[2. Nieto-Castanon A. Handbook of functional connectivity magnetic resonance imaging methods in CONN: Hilbert Press; 2020.](https://web.endnote.com/reference-list/)

[3. Friston KJ, Williams S, Howard R, Frackowiak RS, Turner R. Movement‐related effects in fMRI time‐series. Magnetic resonance in medicine. 1996;35(3):346-55.](https://web.endnote.com/reference-list/)

[4. Power JD, Mitra A, Laumann TO, Snyder AZ, Schlaggar BL, Petersen SE. Methods to detect, characterize, and remove motion artifact in resting state fMRI. Neuroimage. 2014;84:320-41.](https://web.endnote.com/reference-list/)

[5. Hallquist MN, Hwang K, Luna B. The nuisance of nuisance regression: spectral misspecification in a common approach to resting-state fMRI preprocessing reintroduces noise and obscures functional connectivity. Neuroimage. 2013;82:208-25.](https://web.endnote.com/reference-list/)

[6. Behzadi Y, Restom K, Liau J, Liu TT. A component based noise correction method (CompCor) for BOLD and perfusion based fMRI. Neuroimage. 2007;37(1):90-101.](https://web.endnote.com/reference-list/)

[7. Chai XJ, Castañón AN, Öngür D, Whitfield-Gabrieli S. Anticorrelations in resting state networks without global signal regression. Neuroimage. 2012;59(2):1420-8.](https://web.endnote.com/reference-list/)

[8. Nieto-Castanon A. Preparing fMRI data for statistical analysis. arXiv preprint arXiv:221013564. 2022.](https://web.endnote.com/reference-list/)

[9. Rolls ET, Joliot M, Tzourio-Mazoyer N. Implementation of a new parcellation of the orbitofrontal cortex in the automated anatomical labeling atlas. Neuroimage. 2015;122:1-5.](https://web.endnote.com/reference-list/)
